# Cartilage Oligomeric Matrix Protein (COMP) Correlates with Disease Progression, Selected Immune Checkpoint Molecules and SIGLEC9 in Colorectal Cancer

**DOI:** 10.3390/ijms27136032

**Published:** 2026-07-05

**Authors:** Piotr Limanówka, Anna Kot, Wiktor Wagner, Błażej Ochman, Sylwia Mielcarska, Agnieszka Kula, Miriam Dawidowicz, Dorota Hudy, Monika Szrot, Jerzy Piecuch, Zenon Czuba, Elżbieta Świętochowska, Iwona Gisterek-Grocholska, Dariusz Waniczek

**Affiliations:** 1 Department of Medical and Molecular Biology, Faculty of Medical Sciences in Zabrze, Medical University of Silesia, 41-808 Zabrze, Poland; s85876@365.sum.edu.pl (A.K.); s86294@365.sum.edu.pl (W.W.); d201228@365.sum.edu.pl (B.O.); d201109@365.sum.edu.pl (S.M.); dorota.hudy@sum.edu.pl (D.H.); 2 Department of Oncological Surgery, Faculty of Medical Sciences in Zabrze, Medical University of Silesia, 41-808 Katowice, Poland; d201070@365.sum.edu.pl (A.K.); d201069@365.sum.edu.pl (M.D.); dwaniczek@sum.edu.pl (D.W.); 3 Department of General and Bariatric Surgery and Emergency Medicine in Zabrze, Faculty of Medical Sciences in Zabrze, Medical University of Silesia, 41-800 Zabrze, Poland; mszrot@sum.edu.pl (M.S.); jpiecuch@sum.edu.pl (J.P.); 4 Department of Microbiology and Immunology, Faculty of Medical Sciences in Zabrze, Medical University of Silesia, 41-808 Zabrze, Poland; zczuba@sum.edu.pl; 5 Department of Oncology and Radiotherapy, Medical University of Silesia, 40-615 Katowice, Poland; igisterek@sum.edu.pl

**Keywords:** cartilage oligomeric matrix protein, colorectal cancer, tumor microenvironment, tumor-infiltrating lymphocytes, TIM-3, galectin 9, SIGLEC9

## Abstract

Cartilage oligomeric matrix protein (COMP) influences extracellular matrix remodeling. We investigated its clinical, prognostic, and immunomodulatory significance in colorectal cancer (CRC). COMP was quantified via ELISA in 107 paired CRC and normal tissues. Expression was correlated with clinicopathological features, mutational profiles, microsatellite instability (MSI), tumor-infiltrating lymphocytes (TILs), immune checkpoints, and multiplex cytokine networks. For transcriptomic validation, the FieldEffectCrc dataset was used for Gene Set Enrichment Analysis (GSEA), and The Cancer Genome Atlas (TCGA) CRC cohort for survival analysis. COMP was significantly upregulated in CRC tissues (*p* < 0.001) and correlated with advanced T, N, and overall pathological stages (all *p* < 0.05, tau = 0.18, 0.21, and 0.23, respectively). High COMP expression was linked to restricted immune infiltration (reduced stromal TILs, *p* < 0.05, tau = −0.23), elevated levels in microsatellite stable (MSS) compared to MSI tumors (*p* < 0.01), and correlated positively with immune exhaustion markers (T-cell immunoglobulin and mucin-domain containing-3 (TIM-3), galectin-9 (GAL9), sialic acid-binding Ig-like lectin 9 (SIGLEC9)). Transcriptomic data linked high COMP to worse disease-specific and progression-free survival, and enrichment in pro-tumorigenic pathways (epithelial-to-mesenchymal transition, angiogenesis, IL-6 signaling). COMP upregulation defines an immunosuppressive microenvironment in CRC, particularly in MSS tumors. It represents an important prognostic biomarker and potential therapeutic target for overcoming immunotherapy resistance.

## 1. Introduction

Colorectal cancer (CRC) is a growing health problem worldwide. It is the third most common malignancy globally, accounting for approximately 10% of all newly diagnosed cancer cases, and the second leading cause of cancer-related mortality [1].

The unfavorable prognosis in advanced stages is largely driven by intrinsic and acquired therapeutic resistance. The initiation and progression of CRC are driven by a sequential accumulation of genetic and epigenetic alterations. The complex molecular mechanisms underlying colorectal tumorigenesis and progression involve several pathways, including the β-catenin/APC, EGFR/Ras, DCC/SMAD4, and p53, alongside the disruption of essential DNA repair pathways [2]. These molecular alterations not only drive uncontrolled cancer cell proliferation but also dynamically orchestrate interactions with the surrounding tumor. Interestingly, aberrant remodeling and specific changes within the tumor microenvironment (TME) and extracellular matrix (ECM) are recognized as critical drivers of therapeutic resistance, immune evasion, and disease progression [3]. The TME addresses a complex ecosystem of immune cells, cancer-associated fibroblasts, blood vessels, ECM components, and other factors that collectively managing tumor behavior and response to therapy [4,5]. Far from being only structural scaffolding, the ECM actively drives tumor progression through compositional changes, proteolytic degradation, and altered mechanical stiffness in tumor cells [5]. This extensive matrix remodeling creates an immunosuppressive and pro-angiogenic environment that facilitates chemoresistance development and metastatic progression [6,7].

Among the various molecules driving this pro-tumorigenic ECM remodeling, Cartilage oligomeric matrix protein (COMP) has emerged as a particularly important protein. It is an ECM glycoprotein originally identified as a structural component of cartilage [8]. COMP is a member of thrombospondin family expressed mainly in chondrocytes, tenocytes, or myofibroblasts, and plays a role in tissue stabilization by interacting with various ECM components, including collagens I, II, IX, XII, XIV, fibronectin, matrilins-1, 3, 4, and proteoglycans [9,10]. Moreover, COMP regulates the interactions between cells and growth factors [9,11]. It promotes TGF-β activity, takes part in tissue fibrosis driven by TGF-β or biomechanical forces, and modulates the complement system activity [9,12]. It also serves as a marker of tissue destruction and atherosclerosis [9,13]. Apart from its physiological functions, COMP has emerged as a crucial oncogenic factor in multiple tumors, including CRC, breast cancer, gastric cancer and esophageal carcinoma [14,15,16,17]. Mechanistically, COMP promotes tumor aggressiveness by activating the PI3K/Akt/mTOR pathway to enhance cell proliferation, co-expressing with epithelial–mesenchymal transition (EMT) related genes such as *VIM*, *TWIST1*, *ZEB1*, and *MMP9*, and driving cancer cell motility and invasion [18,19].

The structural remodeling of the TME, driven by proteins such as COMP, creates a significant barrier to immune cell infiltration. This phenomenon severely limits the efficacy of modern treatments. While immunotherapy is a novel and highly effective therapeutic approach in CRC, its clinical benefit is currently limited to the approximately 10–15% of patients presenting with microsatellite instability-high (MSI-H) or mismatch repair-deficient (dMMR) tumors [20]. This significant limitation underscores the urgent need to better understand the role of COMP as matrix-modifying molecule in ECM remodeling and in CRC tissues. Better understanding of these mechanisms is essential for predicting treatment efficacy and establishing personalized therapeutic strategies.

To address this clinical challenge, the aim of this study was to evaluate COMP levels in CRC tissues and adjacent normal margins, and to comprehensively investigate their associations with specific clinicopathological parameters, mutational profiles, and immunological features, such as tumor-infiltrating lymphocytes (TILs), microsatellite instability (MSI) status, immune checkpoint proteins, and cytokine networks. Consequently, this approach aims to provide insights into the potential role of COMP in shaping the TME.

## 2. Results

### 2.1. COMP Expression in Colorectal Cancer and Adjacent Normal Tissues

We examined COMP expression in 107 paired tumor tissues and their corresponding adjacent surgical margin samples. To account for the wide range and skewness of protein expression values, data were log10-transformed, which facilitates comparison across samples and stabilizes variance. Statistical parameters, including effect sizes and 95% confidence intervals for all conducted analyses, are summarized in Supplementary Table S1.

**Figure 1:**
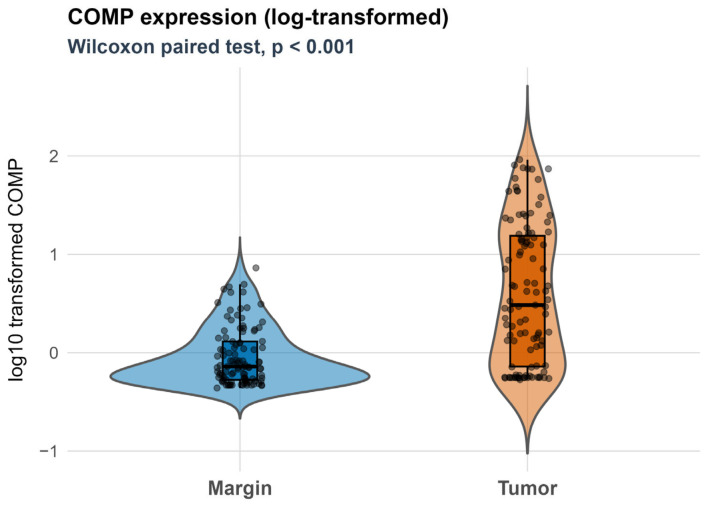
Comparison of COMP protein expression between tumor tissue and matched surgical margins. Abbreviations: COMP, Cartilage Oligomeric Matrix Protein.

In our study, COMP expression was notably higher in tumor tissues, with a median log10-transformed value of 0.49 (Interquartile Range (IQR) 1.33), compared to −0.14 (IQR 0.39) in the matched margin samples. Using a paired Wilcoxon signed-rank test, we confirmed that this difference was highly significant (*p* < 0.001) (Figure 1). These findings indicate that COMP is consistently upregulated in tumor tissue relative to adjacent margins.

### 2.2. Association of COMP Levels with Clinicopathological Features

**Figure 2:**
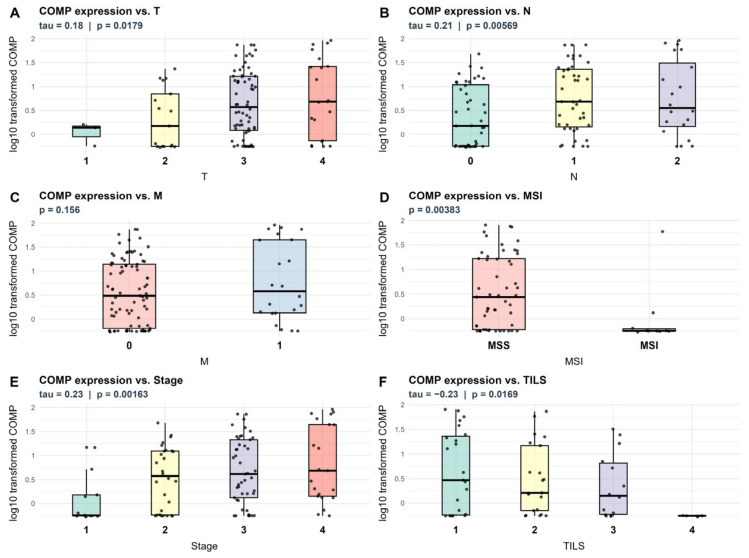
Associations between COMP expression and clinicopathological features of colorectal cancer. (**A**) Tumor size (T), (**B**) lymph node involvement (N), (**C**) presence of metastases (M), (**D**) microsatellite instability (MSI) status, (**E**) tumor stage, and (**F**) stromal tumor-infiltrating lymphocytes (TILs). Data are presented as individual values with median indicated. Abbreviations: COMP, Cartilage Oligomeric Matrix Protein; MSS, microsatellite stable.

We proceeded to investigate the associations between COMP expression and clinical characteristics. Using a Kendall’s tau correlation test, we observed a significant positive association between COMP expression and primary tumor (T) stage (tau = 0.18, *p* = 0.0179), indicating that higher COMP expression tends to be associated with more advanced tumor stages (Figure 2A).

Similarly, for pathologic lymph node (N) stage, Kendall’s tau correlation revealed a significant positive association between COMP expression and N stage (tau = 0.21, *p* = 0.00569), suggesting that higher COMP expression is related to increased lymph node involvement (Figure 2B).

For pathologic distant metastasis (M) stage, a Wilcoxon test comparing COMP expression between these two groups did not reveal any significant differences, indicating that COMP expression was not associated with distant metastasis in this dataset (Figure 2C).

Next, we analyzed the association of COMP expression with MSI status. Comparing samples with microsatellite stable (MSS) (*n* = 53) and MSI (*n* = 10), we observed a clear difference in expression levels. The median log-transformed COMP expression in the MSS group was 0.441 (IQR = 1.45), whereas in the MSI group, it was markedly lower at −0.251 (IQR = 0.0471). A Wilcoxon test confirmed that this difference was statistically significant (*p* = 0.00383), indicating that COMP expression is significantly reduced in tumors with microsatellite instability compared to MSS tumors (Figure 2D).

Considering the overall pathologic stage, Kendall’s tau correlation indicated a significant positive association between COMP expression and overall stage (tau = 0.23, *p* = 0.00163), further supporting the notion that higher COMP concentrations are linked to more advanced disease (Figure 2E).

A multivariable linear regression analysis was performed to evaluate the independent associations of tumor stage, MSI status, and stromal TILs with COMP expression. Although the overall model was significant (*p* = 0.012), individual parameters did not retain independent significance (Stage *p* = 0.086; MSI *p* = 0.234; TILs *p* = 0.264). Variance Inflation Factor (VIF) scores (<1.2) ruled out multicollinearity. This loss of independent significance is possibly attributable to reduced statistical power (model *n* = 61) caused by missing MSI data.

Finally, for TILS, Kendall’s tau correlation revealed a significant negative association with COMP expression (tau = −0.23, *p* = 0.0169), suggesting that higher COMP levels are associated with lower lymphocytic infiltration within the tumor (Figure 2F).

### 2.3. COMP Expression Across Specific Oncogenic Mutation Profiles

Mutation status of the *PIK3CA*, *NRAS*, *BRAF*, *KRAS*, and *AKT* genes was assessed in a subset of our clinical cohort comprising 87 CRC patients. No mutations in the *AKT* gene were detected. Subsequently, we examined whether COMP expression levels differed according to the mutational status of these genes.

**Figure 3:**
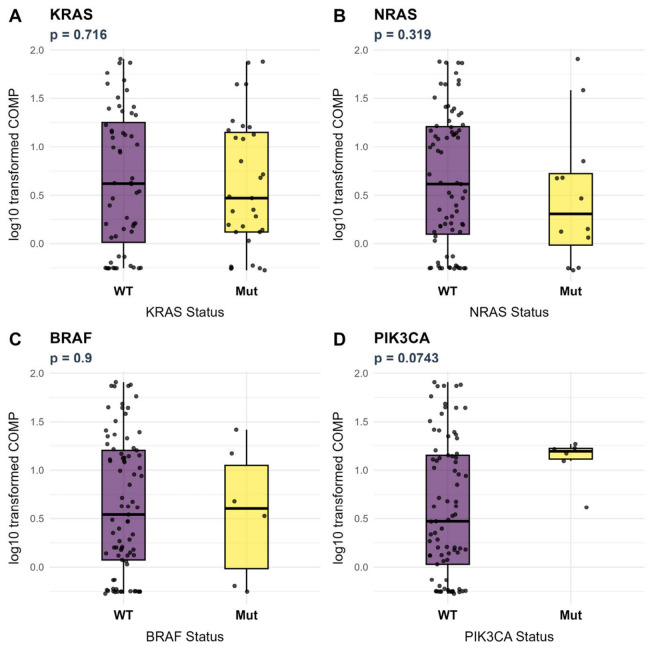
COMP expression in colorectal cancer samples stratified by selected mutations: (**A**) *KRAS*, (**B**) *NRAS*, (**C**) *BRAF*, and (**D**) *PIK3CA*. Abbreviations: COMP, Cartilage Oligomeric Matrix Protein; WT, wild-type; Mut, mutated.

Within our experimental patient group, *PIK3CA* mutations were found in 6 patients (81 wild-type), *NRAS* in 12 (75 wild-type), *KRAS* in 31 (56 wild-type), and *BRAF* in 6 (81 wild-type). Statistical analysis showed no significant differences in COMP expression between mutant and wild-type tumors for any of the analyzed genes (*p* > 0.05) (Figure 3). Overall, COMP expression was not significantly associated with the presence of *PIK3CA*, *NRAS*, *BRAF*, or *KRAS* mutations in CRC. The detailed frequencies of the evaluated gene mutations within our experimental group are summarized in Table 1.

### 2.4. Correlation of COMP with Selected Immune Checkpoint Markers

**Figure 4:**
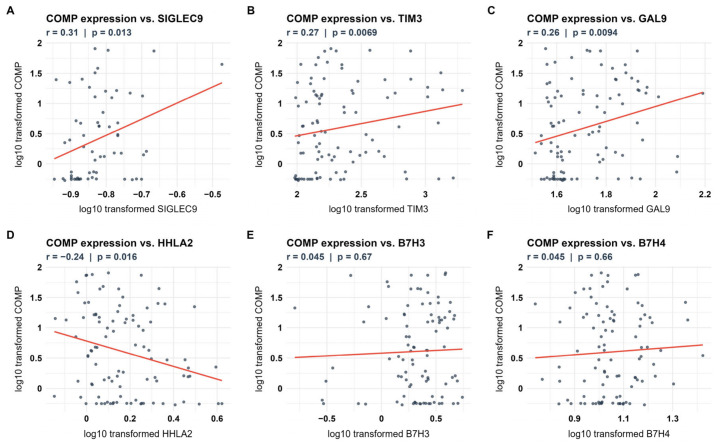
Correlation between COMP expression and the expression of Selected Immune Checkpoint Subset in colorectal cancer samples: (**A**) SIGLEC9, (**B**) TIM3, (**C**) GAL9, (**D**) HHLA2, (**E**) B7H3, and (**F**) B7H4. Abbreviations: COMP, Cartilage Oligomeric Matrix Protein; SIGLEC9, sialic acid-binding Ig-like lectin 9; TIM3, T-cell immunoglobulin and mucin-domain containing-3; GAL9, galectin-9; HHLA2, Human endogenous retrovirus-H long terminal repeat-associating protein 2; B7H3, B7 homolog 3; B7H4, B7 homolog 4.

Expression of six proteins constituting the Selected Immune Checkpoint Subset were measured in tumor tissue samples from 97 CRC patients. Statistically significant correlations were observed between COMP levels and four of these immune markers. Specifically, sialic acid-binding Ig-like lectin 9 (SIGLEC9), T-cell immunoglobulin and mucin-domain containing-3 (TIM3), and galectin-9 (GAL9) showed significant positive correlations with COMP expression (r = 0.31 and *p* = 0.013 for SIGLEC9 (Figure 4A), r = 0.27 and *p* = 0.0069 for TIM3 (Figure 4B), r = 0.26 and *p* = 0.0094 for GAL9 (Figure 4C)). In contrast, Human endogenous retrovirus-H long terminal repeat-associating protein 2(HHLA2) showed a statistically significant negative correlation with COMP levels (r = −0.24 and *p* = 0.016 (Figure 4D)). No significant correlations were observed for B7 homolog 3 (B7H3) or B7 homolog 4 (B7H4) (Figure 4E,F). These findings suggest a potential link between COMP expression and immune-related processes within the TME, possibly reflecting COMP involvement in modulating local immune responses.

### 2.5. Prognostic Value and Clinical Associations of COMP mRNA in the TCGA Cohort

**Figure 5:**
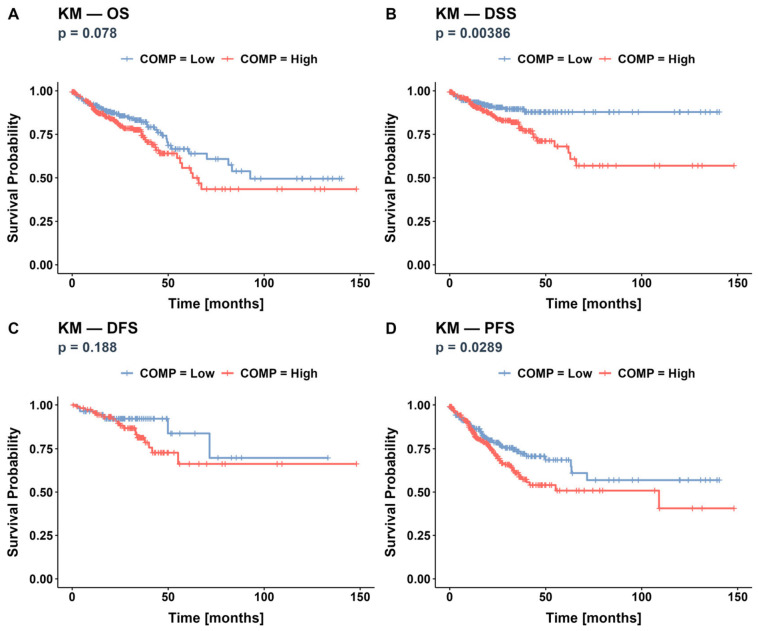
Kaplan–Meier survival analysis of colorectal cancer patients stratified by COMP expression levels. Patients were divided into high and low expression groups based on the median COMP value. Survival outcomes analyzed include (**A**) overall survival (OS), (**B**) disease-specific survival (DSS), (**C**) disease-free survival (DFS), and (**D**) progression-free survival (PFS). Abbreviations: COMP, Cartilage Oligomeric Matrix Protein.

To examine whether the clinical associations and correlations Selected Immune Checkpoint Subset proteins were also reflected at the transcriptomic level, we analyzed RNA expression data from The Cancer Genome Atlas (TCGA) CRC cohort. Patients from the TCGA cohort were stratified into COMP-high and COMP-low groups using the median mRNA expression value as the cutoff. Based on this division, we assessed the prognostic significance of COMP expression across multiple survival endpoints (Figure 5).

Kaplan–Meier survival analysis revealed that patients with high COMP mRNA levels exhibited a trend toward poorer OS, although this difference did not reach statistical significance (*p* = 0.078) (Figure 5A). Notably, elevated COMP expression was significantly associated with worse DSS (*p* = 0.0039) (Figure 5B), indicating a higher risk of cancer-related mortality in this group. While DFS did not differ significantly between the two cohorts (*p* = 0.19) (Figure 5C), patients in the COMP-high group demonstrated significantly shorter PFS (*p* = 0.029) (Figure 5D).

Collectively, these findings explicitly show that elevated COMP mRNA expression is linked to an unfavorable clinical course, particularly contributing to disease progression and cancer-specific mortality in CRC.

**Figure 6:**
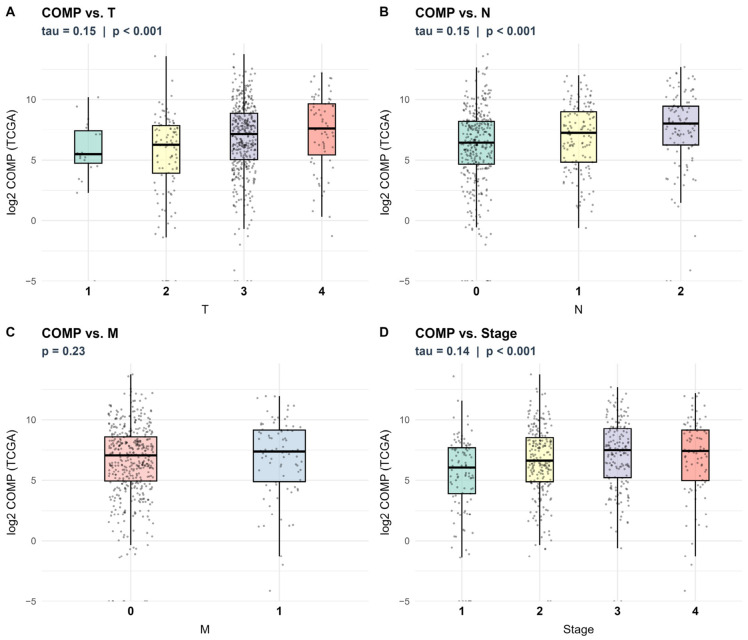
COMP mRNA expression stratified by clinicopathological features: (**A**) T stage, (**B**) N stage, (**C**) M status, and (**D**) overall pathological stage. Abbreviations: COMP, Cartilage Oligomeric Matrix Protein; T, primary tumor; N, regional lymph node; M, distant metastasis; TCGA, The Cancer Genome Atlas.

Next, we investigated whether COMP mRNA expression was associated with clinical and pathological features in the TCGA CRC cohort. We evaluated associations between COMP expression and T, N, M status, as well as the overall pathological stage. Using Kendall’s tau correlation test, COMP expression was positively associated with T stage, N stage, and overall pathological stage (all *p* < 0.001, Kendall’s tau = 0.15, 0.15, and 0.14, respectively). In contrast, no significant differences were observed for M status based on the Wilcoxon test (Figure 6).

These findings are consistent with observations from our patient cohort, confirming that COMP expression both at the protein and RNA levels is linked to disease progression and demonstrates prognostic relevance in CRC.

### 2.6. Transcriptomic Validation of COMP-Immune Checkpoint Correlations

Next, we aimed to determine whether the correlations observed in our patient cohort at the protein level were also present at the RNA level in the TCGA CRC cohort. To this end, Spearman correlation analyses were performed between log2-transformed expression of genes corresponding to Selected Immune Checkpoint Subset proteins and COMP.

**Figure 7:**
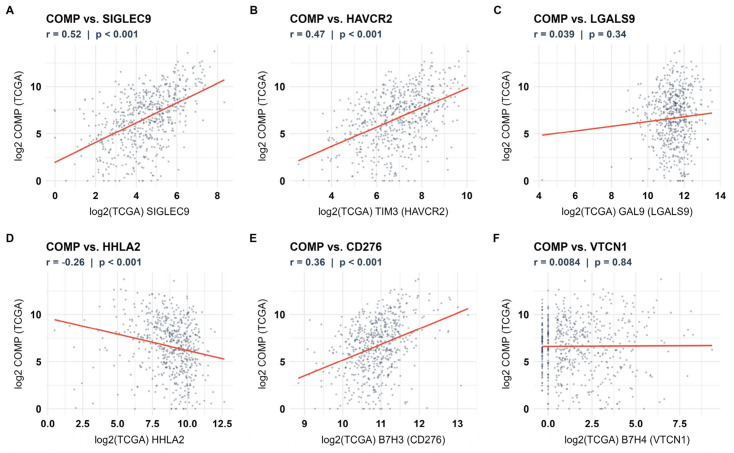
Correlation of COMP mRNA expression with Selected Immune Checkpoint Subset genes in colorectal cancer samples. Panels show associations with (**A**) *SIGLEC9*, (**B**) *HAVCR2*, (**C**) *LGALS9*, (**D**) *HHLA2*, (**E**) *CD276*, and (**F**) *VTCN1*. Abbreviations: COMP, Cartilage Oligomeric Matrix Protein; TCGA, The Cancer Genome Atlas; *SIGLEC9*, sialic acid-binding Ig-like lectin 9; *HAVCR2*, hepatitis A virus cellular receptor 2 (TIM-3); *LGALS9*, galectin-9; *HHLA2*, Human endogenous retrovirus-H long terminal repeat-associating protein 2; CD276, B7 homolog 3; VTCN1, V-set domain-containing T-cell activation inhibitor 1 (*B7-H4*).

The results partially reflected our protein-level observations. Specifically, *SIGLEC9*, *TIM3*, and *HHLA2* showed similar patterns as in our cohort, with statistically significant correlations with COMP expression (*p* < 0.001 and r = 0.52, 0.47, and −0.26, respectively, for *SIGLEC9*, *TIM3*, and *HHLA2*). In contrast, no significant correlation was observed for *GAL9*, which had shown a positive association with COMP at the protein level in our cohort. Interestingly, *B7H3* displayed a significant positive correlation in TCGA (*p* < 0.001 and r = 0.36), whereas no significant relationship had been observed in our cohort. *B7H4*, similar to our protein-level analysis, did not show a significant correlation with COMP expression in TCGA (Figure 7A–F). Overall, these findings confirm several of the correlations identified in our cohort, but also suggest possible post-transcriptional regulation of specific genes, as their relationships with COMP differ between protein and RNA levels.

### 2.7. PCA-Based Assessment of COMP-Associated Cytokine and Immune Regulatory Networks

To further explore the associations between COMP expression and the immune TME within our clinical cohort, we performed Principal Component Analysis (PCA) on Selected Immune Checkpoint Subset molecules and functionally grouped cytokine sets. In the analysis of Selected Immune Checkpoint Subset, the first two Principal Components (PCs) accounted for 61.48% of the total variance. The PC1 dimension was primarily driven by strong positive loadings of TIM-3 and GAL9. Meanwhile, PC2 captured B7-H3 and B7-H4 with positive loadings, opposed by strong negative contributions from SIGLEC9, CTLA4, and HHLA2. Linear regression revealed a highly significant positive correlation between COMP expression and PCA Factor 1 (R = 0.6484, *p* = 0.001985), indicating a coordinated upregulation of a TIM-3/GAL9-driven checkpoint regulation in COMP-high tumors (Figures S1–S3, Tables S2 and S3).

We subsequently analyzed the relationship between COMP expression and specific cytokine signaling pathways, identifying number of significant associations with distinct cytokine subsets. For the Interleukin receptor SHC signaling network (Reactome HSA-912526), PC1 explained 77.48% of the variance and was dominantly driven by IL-3, IL-2, GM-CSF, and IL-5. However, COMP expression showed a significant negative association (R = −0.4595, *p* = 0.04154) specifically with PCA Factor 2, which was primarily characterized by the negative contribution of IL-2Ra (Figures S4–S6, Tables S4 and S5).

Similar inverse correlations were observed for specific functional axes within other immune regulatory networks. COMP expression correlated negatively with the PC2 of Interleukin-12 signaling (Reactome HSA-9020591; Factor 2: R = −0.5128, *p* = 0.02077), a dimension characterized by opposing contributions of IFN-γ and MIF (Figures S7–S9, Tables S6 and S7). Analysis of cytokines involved in the regulation of interleukin-6 production (GO:0032675) revealed a positive correlation between COMP expression and PCA Factor 2 (R = 0.4381, *p* = 0.05). This specific component described a distinct proinflammatory profile driven by strong positive contributions from HGF, IL-16, and IL-6, opposed by IFN-γ and IL-1β (Figures S10–S12, Tables S8 and S9). Furthermore, COMP concentrations were negatively associated with PCA Factor 3 of the Regulation of interleukin-12 production subset (GO:0032655; R = −0.4945, *p* = 0.02666), which was strongly driven by IL-17, and with PCA Factor 3 of the Th17 cell differentiation network (KEGG hsa04659; R = −0.5715, *p* = 0.008482), an axis mainly defined by positive loadings of IL-1β and IL-17 (Figures S13–S15, Tables S10 and S11). The significant associations between COMP concentration and the PCs representing the identified immune-related subsets are summarized in Table 2.

### 2.8. GSEA of Transcriptomic Alterations Associated with COMP Expression

**Figure 8:**
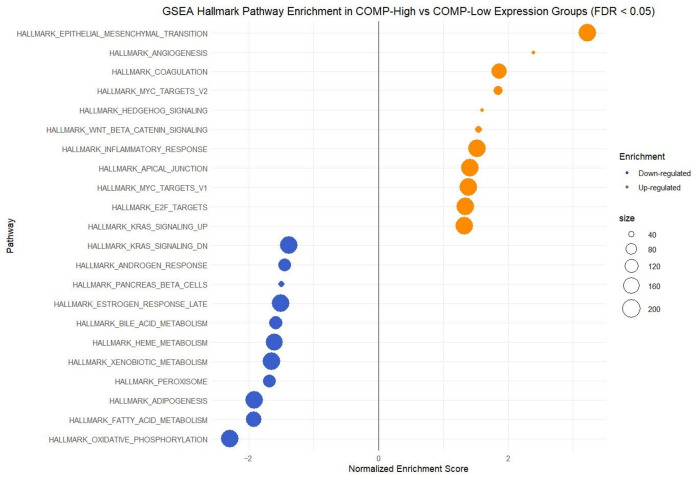
GSEA of Hallmark pathways in COMP-high versus COMP-low tumors (FDR < 0.05). The x-axis represents the normalized enrichment score (NES). Orange circles indicate pathways enriched in COMP-high samples, whereas blue circles indicate pathways enriched in COMP-low samples. Circle size reflects gene set size. Abbreviations: GSEA, Gene Set Enrichment Analysis; COMP, Cartilage Oligomeric Matrix Protein; FDR, false discovery rate; NES, normalized enrichment score.

To gain broader mechanistic insights into the transcriptomic alterations associated with COMP mRNA levels, we performed GSEA on Hallmark pathways using the FieldEffectCrc dataset, comparing COMP-high and COMP-low tumor groups. Tumors with high COMP expression showed significant enrichment (false discovery rate (FDR) < 0.05) in pathways promoting tumor aggressiveness and immune modulation, including EMT (normalized enrichment score (NES) = 3.23), Angiogenesis (NES = 2.38), Coagulation (NES = 1.87), and Inflammatory Response (NES = 1.53) (Figure 8, Table S14). In contrast, tumors characterized by low COMP expression exhibited a strong enrichment in various metabolic programs. The most significantly downregulated pathways in COMP-high samples included Oxidative Phosphorylation (NES = −2.29), Fatty Acid Metabolism (NES = −1.92), and Adipogenesis (NES = −1.91) (Figure 8, Table S14). These findings suggest that high COMP expression is linked to a major metabolic shift and enhanced pro-tumorigenic, mesenchymal, and inflammatory signaling within the CRC microenvironment.

## 3. Discussion

COMP is an ECM glycoprotein implicated in a variety of pathological processes associated with both cellular functions and changes in ECM [21]. Notably, COMP exhibits increased expression in various malignancies, and has been proposed as an important biomarker for numerous cancer types [22,23]. In CRC, it has been shown to facilitate tumorigenesis and disease progression by promoting cancer cell proliferation, EMT and metastasis, partly through the activation of the PI3K/AKT pathway [18,19,24,25]. Its expression is especially elevated in early-onset colon cancer [18,25], which tends to take a more aggressive course and features high levels of chromosomal instability compared to the disease developing in older patients [26]. Consistently, higher COMP expression in CRC is often associated with unfavorable prognosis [25,27,28]. COMP upregulation in CRC correlates positively with advanced TNM stage, worse grading and increased tumor fibrosis [27]. Furthermore, it is significantly associated with worse OS, DSS, and recurrence-free survival (RFS) [28]. In this study, we evaluated the clinical, transcriptomic, and immunological landscape of COMP in CRC, revealing its significant upregulation and association with disease progression and an immunosuppressive TME.

In line with previous findings [23,29], we showed that COMP was markedly upregulated in CRC specimens compared to the adjacent surgical margins. Moreover, we demonstrated that COMP levels correlated with higher T stage, N stage, and overall pathological stage, highlighting its association with local tumor progression and regional dissemination. These clinical associations are largely consistent with the study by Blom et al., who linked elevated COMP expression to TNM parameters [27].

However, neither our protein-level analysis nor our evaluation of COMP mRNA in the TCGA CRC cohort revealed a significant correlation with distant metastasis (M stage). While existing transcriptomic reports support our findings [20,23], Blom et al. found a statistically significant association between COMP staining intensity in CRC tissues and all parameters of the TNM scale, including distant metastases, as well as the tumor grade [27]. The lack of significance in our cohort (*p* = 0.156) might be attributed to different evaluation techniques and the limited number of patients presenting with metastatic disease at surgery (M1, *n* = 22 vs. M0, *n* = 85), reducing the statistical power to detect this specific association.

Notably, various studies identify elevated COMP as an indicator of worse prognosis in CRC and other malignancies [22]. High COMP RNA expression correlates with OS, DFS, and PFI (progression-free interval) [20,23]. Furthermore, Blom et al. demonstrated an association between strong immunohistochemical staining for COMP and shorter OS [27].

In our transcriptomic analysis of the TCGA CRC cohort, high COMP mRNA expression was significantly associated with shorter disease-specific survival (DSS) and progression-free survival (PFS). Although our data demonstrated only a trend toward significance for overall survival (OS), this endpoint-specific finding could highlight the precise prognostic value of COMP. Overall survival in CRC is confounded by all-cause mortality, which is a critical factor given the typically advanced age and comorbidities of this patient demographic. In contrast, DSS serves as a direct measure of mortality attributable to the underlying malignancy. The statistical significance observed exclusively for DSS and PFS demonstrates that the adverse prognostic impact of COMP is likely tied to direct tumor progression and cancer-specific mortality, rather than general patient frailty or unrelated causes of death.

Various reports demonstrate that COMP-mediated remodeling increases ECM stiffness, possibly driving pathological processes within the TME [14,23,30]. This matrix is primarily shaped by cancer-associated fibroblasts (CAFs). In malignancies, aberrant collagen rearrangement and excessive protein secretion by CAFs lead to increased stromal stiffening. Signaling axes involving TGF-β, TNF-α, and PI3K/Akt further accelerate this process. Consequently, the resulting fibrosis creates a physical barrier that restricts drug delivery and limits immune cell infiltration, while simultaneously providing mechanical signals that promote tumor progression [31].

COMP expression is directly modulated by TGF-β, and high COMP levels identify tumors with active TGF-β signaling [23,30]. Elevated COMP levels in the CRC stroma correlate positively with tumor fibrosis and classical CAF markers including α-SMA, fibroblast activation protein, S100A4, vimentin, and fibronectin. This fibrotic environment leads to poorer overall immune cell infiltration [27], as these physical barriers are built primarily by extracellular COMP secreted by CAFs [30]. Consistent with this mechanism, our clinical cohort analysis demonstrated a significant negative association between COMP expression and stromal tumor-infiltrating lymphocytes. Notably, lower levels of TILS are an indicator of worse prognosis in CRC [32]. The literature presents mixed evidence regarding immune infiltration, which likely results from the varying effects of COMP on specific immune cell populations. Some authors describe a negative correlation between COMP expression and bulk TILs or effector CD8 T cells [20,27,33]. However, Ding et al. reported a positive association with total immune cells [23], and Ma et al. demonstrated increased infiltrations of CAFs and M2 macrophages in tumors with high COMP levels [20]. These differences suggest a potential dual immunomodulatory role for COMP. ECM remodeling induced by COMP may physically restrict the access of anticancer effector cells. At the same time, this altered stroma could facilitate the recruitment and activity of immunosuppressive cells, specifically M2 macrophages and exhausted T cells. Overall, COMP likely contributes to a pathological TME that limits anti-tumor immunity while promoting ECM remodeling, angiogenesis, and cancer cell migration.

COMP also interacts with immune checkpoint pathways. Ding et al. reported positive correlations between COMP and various immunomodulatory genes, including *HAVCR2*, across multiple cancers [23]. However, Blom et al. linked COMP to decreased PD-L1 expression [27]. In our clinical cohort, COMP concentration positively correlated with SIGLEC9, TIM3, and GAL9, while correlating negatively with HHLA2. Our TCGA transcriptomic analysis largely confirmed these patterns, though specific differences emerged, such as the lack of GAL9 mRNA correlation. This discordance between mRNA and protein levels may arise from several potential factors, including post-transcriptional regulation, differences in protein half-lives, or dynamic proteolytic shedding within the TME. Furthermore, PCA revealed a significant positive correlation between COMP expression and a specific dimension driven by TIM-3 and GAL9. This coordinated upregulation points to a functional link between COMP and the TIM-3/GAL9 checkpoint axis. Similarly to COMP, TGF-β can upregulate both TIM-3 and GAL9. The TIM-3/GAL9 pathway has been found to promote TNF-α signaling, facilitate tumor growth, and silence anti-tumor responses [34]. Furthermore, GAL9 has been implicated in the resistance to anti-PD1 strategies [35]. Consequently, the accumulation of COMP likely correlates with an immunosuppressive landscape that drives resistance to standard immunotherapies, including anti-PD-(L)1 treatments. However, these findings should be interpreted with caution, given the observed inconsistencies in our results.

Analysis of cytokine signaling pathways revealed distinct transcriptomic shifts associated with COMP expression. PCA demonstrated a significant negative correlation between COMP and dimensions driven by IL-1β and IL-17, which modulate the IL-12 axis and Th17 cell differentiation. IL-1β, secreted by various cell populations, including fibroblasts, immune cells or cancer cells, upon the interaction with its receptors, activates the p38/JNK and NF-κB pathways, regulating gene transcription. Interestingly, this proinflammatory cytokine can exert both anti- and protumor effects, likely depending on cellular context. Still, IL-1β supports tumorigenesis through numerous action mechanisms, promoting angiogenesis, cancer cell proliferation, and invasion [36]. Notably, IL-1β has been shown to abrogate TGF-β1-induced COMP synthesis in chondrocytes and synovial cells [37]. Moreover, IL-1β supports COMP degradation through ADAMTS-7 and ADAMTS-12 [38]. These reports are consistent with the negative association between COMP and the IL-1β-driven dimension in our study. IL-12, on the other hand, is a proinflammatory cytokine, contributing to enhanced cytotoxic T-cell and NK cell function, the inhibition of immunosuppressive cells and induction of IFN-γ secretion [39]. Altering the IL-12 signaling could be another mechanism in which COMP diminishes immune responses in TME. Additionally, while IL-17 plays a complex role in tumorigenesis, it frequently serves as a marker for a favorable clinical course in CRC [40]. Aligning with our PCA results, Ding et al. previously showed that COMP expression negatively correlates with Th17 cell infiltration [23]. In contrast, COMP positively associated with a proinflammatory component driven by HGF, IL-16, and IL-6. IL-16 has been reported to promote tumor growth, while HGF signaling has been implicated in disease progression, EMT, angiogenesis and metastasis [41,42]. The IL-6 pathway actively promotes cancer cell proliferation, angiogenesis, immune evasion, and therapy resistance [43]. The promotion of pathways related to IL-6, IL-16 and HGF could be another factor supporting tumor growth in the case of COMP upregulation, standing in line with the association between COMP and more advanced CRC stage that we found. Moreover, as the mentioned cytokines support ECM remodeling, their promotion could further contribute to altered TME and CRC progression upon COMP upregulation. Together, these findings indicate that COMP is associated with disease progression by shifting the cytokine balance within the TME. This immunomodulation may arise from direct receptor interactions or COMP-mediated ECM remodeling, which provides altered mechanical stimulation to resident immune cells [14,21,30,31]. Importantly, while this matrix remodeling depends mainly on extracellular COMP secreted by CAFs [30], intracellular COMP within cancer cells may independently drive chemotherapy resistance through distinct pathways [44].

Transcriptomic profiling via GSEA provided a broader context for our findings, revealing a metabolic and immunological shift in COMP-high tumors. Rather than pointing to isolated pathways, the data showed enrichment in angiogenesis, hypoxia-related signatures, and EMT. This distinct molecular profile reflects a highly aggressive and remodeled TME, which provides a rationale for the clinical link between COMP and advanced disease stages. The role of COMP in EMT, cancer cell migration and invasion has been supported in other studies [14,20,30]. Additionally, Huang et al. demonstrated that COMP likely modulates EMT-related gene expression through transcriptional regulation [14], while another study suggested the mechanism involving the activation of the Notch3 receptor and β-catenin pathway, promoting cancer cell migration [30]. Interestingly, Wusterbarth and colleagues found that COMP may serve as a biomarker for CRC molecular subtypes. Despite all subtypes exhibiting increased COMP mRNA expression compared to margins, the lowest COMP expression was detected in the epithelial/metabolic Consensus molecular subtypes (CMS) 3 subtype, while the mesenchymal CMS4 subtype exhibited the highest COMP RNA levels [28]. CMS4 tumors are characterized by the enrichment in EMT-related genes, TGF-β signaling, angiogenesis, and matrix remodeling pathways [45]. Peak COMP expression within the mesenchymal CMS4 subtype reinforces the association of COMP with tumor stroma and extensive ECM remodeling [28,45]. Together, these reports confirm that COMP is involved in the alteration of immune landscape in CRC, including the modulation of cytokine signaling and IC expression revealed in our study, likely through the restructuration of ECM.

In CRC, the presence of MSI or MSS directly reflects the functional status of the tumor’s DNA mismatch repair MMR system. dMMR, leading to the MSI phenotype, is biologically characterized by a high mutational rate, which typically elicits a robust local immune response and renders these tumors sensitive to immune checkpoint inhibitors. Conversely, proficient MMR (pMMR) tumors, which exhibit the MSS phenotype, are generally considered immunologically “cold”. They feature limited neoantigen presentation, poor T-cell infiltration, and inherent resistance to current immunotherapies [46]. Interestingly, our results demonstrated elevated COMP expression in MSS CRC compared to MSI disease, supporting previous observation by Ma and colleagues [20]. This implies that COMP could limit immune responses primarily in MSS tumors, contributing to the mechanical barrier between TME and immune system. Similarly, COMP could limit immunotherapy efficacy in MSS CRC. This association between COMP and MSS CRC supports the relations between higher COMP expression, lower immune infiltrations and immunological pathways promoting tumor growth and progression revealed in our study, as MSS CRC is characterized by disrupted anti-cancer immune responses [47]. On the other hand, a different study demonstrated that strong COMP staining in stroma was associated with microsatellite instability [27]. This difference however may result from distinct study methodologies. Blom et al. evaluated spatial protein accumulation using immunohistochemistry, whereas our study assessed average expression profiles across bulk tissue homogenates at both the protein and RNA levels. This contrast suggests that intracellular and stromal COMP may reflect distinct biological processes and present different immunomodulatory effects in the context of MSI. However, our results regarding the association between the MSI status and COMP expression should be interpreted with caution due to a small number of patients with available MSI status included in the study. Altogether, COMP should be considered as an attractive immunotherapeutic target and a potential marker for immunotherapy resistance. Targeting COMP could be especially beneficial in MSS tumors with low immunotherapeutic sensitivity.

The elements of ECM, such as COMP, play a potent role in tumorigenesis. However, the therapeutic potential regarding the ECM modulation strategies in cancer treatment remains unclear. Attempts have been made for developing anti-tumor therapies targeting TGF-β, thus reducing ECM deposition, EMT or CAF development—mechanisms overlapping with COMP action in TME [48]. The preclinical studies regarding TGF-β signaling blockade in cancer have yielded promising results [49]. Similarly, targeting COMP-related pathways could be effective in anti-tumor treatment, and requires in-depth investigation. Moreover, TGF-β signaling targeting could exert its effects partly via COMP. Additionally, the approaches targeting ECM stiffness—including blockers of proteins involved in collagen production, integrins, lysyl oxidases, ion channels, or YAP/TAZ signaling—emerge as novel strategies in cancer therapy [50].

Nevertheless, our study has certain limitations. Our analyses were based on a cohort of 107 paired colorectal cancer and surgical margin tissues. Therefore, some of the broader associations presented herein may require confirmation in larger populations. More importantly, our statistical power to detect subtle correlations was limited in specific subgroups. For instance, MSI status data were available for only 63 patients (with just 10 MSI cases), and the evaluated gene mutations were similarly represented by small patient subsets. The missing MSI data for 44 patients is due to the fact that MSI status was not routinely assessed for a portion of the cohort. This limits our ability to definitively rule out associations within these specific parameters. In the multivariable analysis, individual clinical parameters did not retain independent significance. While VIF assessment indicated a lack of multicollinearity, this outcome is likely constrained by the reduced sample size of patients with complete MSI data (*n* = 61). Further studies in larger cohorts are needed to clarify these independent relationships. Additionally, the results revealed by PCA and GSEA regarding immune pathways associated with COMP may serve only as an indicator, and cannot provide the reader with direct mechanisms of COMP function in TME. Namely, it remains vague whether COMP upregulation is the cause of the observed changes in CRC immune landscape, or whether COMP overexpression results from these alterations in TME, such as the shift in cytokine expression. Moreover, the exact cellular source of COMP could not be determined in our study, as we evaluated only the overall COMP expression in CRC tissues. Altogether, we have demonstrated that changes in COMP expression reflect the restructuration of TME in CRC towards the inhibition of anti-tumor responses. Future studies could shed more light on the subject of COMP in tumor progression, its roles in TME and its therapeutic potential, especially in the context of patient prognosis, MSS-CRC or therapy resistance.

## 4. Materials and Methods

### 4.1. Study Group Characteristics

A total of 214 tissue samples were collected from patients diagnosed with CRC, comprising 107 tumor tissues and 107 matched adjacent surgical margin samples. All cases were histopathologically confirmed by experienced pathologists prior to inclusion in the study.

Only patients who provided written informed consent, were over 18 years of age, and had histologically confirmed colorectal tumors with clear resection margins were included. Patients who did not meet these criteria were excluded from the study.

The study cohort consisted of 57 males and 50 females, with a mean age of 64.65 ± 9.38 years (range: 39–88), with the majority of tumors being located in the left side (63.6%). The study was approved by the Research Ethics Committee (PCN/0022/KB1/42/VI/14/16/18/19/20).

According to the TNM classification, 13 patients (12%) were diagnosed with stage I, 28 (26%) with stage II, 45 (42%) with stage III, and 21 (20%) with stage IV disease. Lymph node involvement (N1, N2) was present in 63 patients (59%), while distant metastases (M1) were observed in 22 patients (21%). The detailed clinicopathological characteristics of the study cohort are summarized in Table 3.

All tissue samples were collected immediately after surgical resection, snap-frozen in liquid nitrogen, and stored at −80 °C until further molecular analyses. The exact number of samples used for each specific analysis is summarized in Table S15.

### 4.2. Mutational Status

Genomic alterations in *KRAS*, *NRAS*, *BRAF*, *PIK3CA*, and *AKT1* genes were analyzed by RT-PCR using the CRC-RT48 Mutation Detection Panel (EntroGen, Woodland Hills, CA, USA). The analysis was carried out following previously established protocols [51].

Genomic DNA was extracted from fresh-frozen CRC tissues stored at −80 °C with an automated nucleic acid isolation system using the Mag-Bind Blood and Tissue DNA HDQ 96 Kit (M6399-00, Omega Bio-tek, Norcross, GA, USA). DNA quantity and purity were assessed spectrophotometrically, and samples were adjusted to 2 ng/μL prior to amplification, as recommended by the manufacturer.

RT-PCR amplification and fluorescence signal detection were carried out on a QuantStudio™ 5 Real-Time PCR System (Thermo Fisher Scientific, Waltham, MA, USA). The assay targets hotspot regions within *KRAS* (exons 2–4), *NRAS* (exons 2–4), *BRAF* (exon 15), *PIK3CA* (exons 9 and 20), and *AKT1* (exon 4). This approach enables sensitive detection of clinically relevant variants, including *KRAS* codons 12/13, 59, 61, 117, 146, *NRAS* codons 12/13, 59, 61, 117, 146, *BRAF* V600, *PIK3CA* codons 542/545 and 1047, and *AKT1* E17K.

### 4.3. Protein Expression Quantification

The concentration of COMP was measured using a Human Cartilage Oligomeric Matrix Protein ELISA kit (Cat. No. RD194080200; Lot No. E24-022; BioVendor—Laboratorní medicína a.s., Brno, Czech Republic) in accordance with the manufacturer’s instructions. Preliminary titration assays were performed to determine the optimal sample dilution. The assay sensitivity was reported to be 0.4 ng/mL. All obtained values were normalized to the total protein content and expressed as ng of COMP per mg of total protein.

The concentrations of specific immune-related proteins within this patient cohort have been previously quantified and reported by our research team. TIM-3 and Gal-9 levels were measured with SEH930Hu and SEA309Hu kits (Cloud Clone, Wuhan, China), respectively, with detection limits of <31 pg/mL and <33 pg/mL, respectively [52]. B7H3 concentration was determined using a human B7H3 ELISA kit (Cloud Clone, Wuhan, China; sensitivity 0.118 ng/mL), whereas HHLA2 concentrations were measured with a human HHLA2 ELISA kit (EIAAB Science Inc., Wuhan, China; sensitivity 0.14 ng/mL) [53,54]. SIGLEC9 concentration was quantified using the SED922Hu kit (Cloud Clone, Wuhan, China, sensitivity 0.057 ng/mL), and B7H4 levels were assessed with a human B7H4 ELISA kit (Cloud Clone, Wuhan, China, sensitivity 56 pg/mL) [51,55]. All spectrophotometric measurements were executed utilizing a μQuant microplate reader (BioTek Instruments, Winooski, VT, USA).

### 4.4. MSI Status Determination

MSI was analyzed in formalin-fixed, paraffin-embedded (FFPE) CRC tissues using immunohistochemistry (IHC), as described previously [55]. The study included 63 tumor samples with assessed MSI status.

Microtome-sectioned tissue specimens (4 μm thickness) were subjected to an automated immunostaining protocol utilizing the Dako Autostainer Link 48 platform (Agilent Technologies, Santa Clara, CA, USA). Following standard deparaffinization and rehydration steps, epitope unmasking was achieved via thermal processing (95 °C for 20 min) immersed in a high-pH EnVision FLEX Target Retrieval Solution (Dako Denmark A/S, Glostrup, Denmark). After blocking endogenous activity, the slides were incubated with monoclonal antibodies targeting mismatch repair (MMR) proteins: MLH1 (clone G168-728, Cell Marque, Rocklin, CA, USA 1:100, 40 min), PMS2 (clone MRQ-28, Cell Marque, 1:50, 40 min), MSH2 (clone G219-1129, Cell Marque, 1:400, 30 min), and MSH6 (clone 44, Cell Marque, 1:100, 45 min).

Detection was carried out using the EnVision FLEX HRP visualization system (Dako) with 3,3′-diaminobenzidine (DAB) as the chromogen. Following hematoxylin counterstaining, slides were washed and mounted for microscopic evaluation.

Expression of MMR proteins was assessed based on nuclear staining in tumor cells. Stromal and inflammatory cells present within each section served as internal positive controls. Loss of nuclear signal in any of the MLH1/PMS2, PMS2 alone, MSH2/MSH6, or MSH6 alone patterns was interpreted as microsatellite instability (MSI-positive) phenotype.

### 4.5. Multiplex Cytokine Profiling and Principal Component Analysis

Homogenized CRC tissue samples (*n* = 54) were analyzed for cytokine, chemokine, and growth factor concentrations using the Bio-Plex Pro Human Cytokine Screening Panel, 48-Plex (Bio-Rad Laboratories, Hercules, CA, USA). All measurements were conducted according to the manufacturer’s protocol. Concentrations obtained from the multiplex assay were normalized to the total protein content of each CRC homogenate. Subsequently, the quantified molecules were assigned to functional categories based on Gene Ontology (GO), Kyoto Encyclopedia of Genes and Genomes (KEGG) and Reactome pathway annotations (Table 4) [56,57,58]. The evaluated pathways include those from a previous study [52], along with new ones added for this analysis. To explore patterns within the dataset, log10-transformed and normalized expression values were subjected to PCA. The covariance matrix was decomposed to derive eigenvalues and eigenvectors, and the first three principal components (PCs) were retained for interpretation. To facilitate clearer separation of variable contributions, varimax orthogonal rotation was applied to the component loadings.

Associations between PC scores and COMP levels were evaluated using Spearman’s rank correlation test, given the non-normal distribution of the data. Statistical significance was set at *p* < 0.05. All computations and visualizations were performed in RStudio (v4.4.1) with the “factoextra” package.

### 4.6. GSEA

Gene Set Enrichment Analysis (GSEA) was carried out in RStudio (v4.4.1) using data from the “FieldEffectCrc” cohort, focusing on CRC samples from Cohort A [59]. The dataset included 834 human colorectal tissue samples. Tumor tissue, adjacent mucosa, and healthy controls with transcript-level quantifications were generated using Salmon. Counts were obtained by summarizing transcript abundances through the tximport package.

For this analysis, only tumor-derived CRC samples (*n* = 311) were retained. Differential expression was computed using the DESeq function from the DESeq2 package, which performs internal normalization for sequencing depth and library size. Normalized gene counts were extracted and used for downstream enrichment testing.

Samples were categorized according to COMP (ENSG00000105664) expression levels. The cohort was divided into “high” and “low” expression groups based on the median normalized expression value.

Gene annotation was performed using org.Hs.eg.db, and genes were ranked according to their differential expression statistics for subsequent GSEA. Enrichment was assessed against the Hallmark gene sets from the Molecular Signatures Database (MSigDB) [60,61] using the fgsea algorithm, with 10,000 permutations and inclusion limits of 15–400 genes per set. Pathways with adjusted *p*-values below 0.05 were considered significantly enriched and ranked according to normalized enrichment score (NES).

### 4.7. TILS

The extent of tumor-infiltrating lymphocytes (TILs) was evaluated semi-quantitatively on hematoxylin and eosin (H&E) stained slides. We applied a five-level scoring system based on the Salgado criteria, which were initially designed for breast tumors [62]. The assessment was strictly limited to the stromal compartment, encompassing both the central stroma and the invasive margin. Tissue areas occupied by cancer cells, along with any inflammatory infiltrates located outside the tumor boundaries, were strictly excluded from the analysis. The final scores reflected the percentage of the stromal area infiltrated by lymphocytes: score 1 for <5%, score 2 for 5–25%, score 3 for 25–50%, score 4 for 50–75%, and score 5 for >75%.

### 4.8. TCGA Dataset and Survival Analysis

To evaluate the transcriptomic landscape and clinical significance of COMP in CRC, clinical and RNA sequencing (RNA-seq) data were acquired from the Colorectal Adenocarcinoma (TCGA, PanCancer Atlas) dataset. Data accession was conducted via the cBioPortal for Cancer Genomics (http://www.cbioportal.org (accessed on 24 October 2025)) [63,64]. The PanCancer Atlas integrates data from the TCGA Colon Adenocarcinoma (COAD) and Rectum Adenocarcinoma (READ) projects. For transcriptomic analyses, batch-normalized mRNA expression values (RNA Seq V2 RSEM) were downloaded and log2-transformed to normalize distributions and stabilize variance.

Matched clinical annotations were queried to extract survival data, including overall survival (OS), disease-specific survival (DSS), disease-free survival (DFS), and progression-free survival (PFS). For survival analyses, the TCGA cohort was stratified into COMP-high and COMP-low expression groups based on the median log2-transformed mRNA expression value. Survival data were available for 588 patients for OS and PFS, 567 patients for DSS, and 222 patients for DFS. Survival probabilities across multiple endpoints were estimated using the Kaplan–Meier method, and statistical differences between the survival curves were evaluated utilizing the log-rank test.

### 4.9. Statistical Analysis

Data distributions were initially evaluated for normality using the Shapiro–Wilk test. Variables were log10-transformed when necessary to improve normality and stabilize variance. Differences in COMP expression between tumor tissue and matched surgical margins were assessed using the Wilcoxon signed-rank test, a non-parametric method suitable for paired samples without assuming normality.

Associations between protein concentrations and TNM staging parameters were analyzed using Kendall’s Tau, while relationships between protein levels and mutation status of the analyzed genes were evaluated with the Mann–Whitney U test. Spearman’s rank correlation was applied for all other correlation analyses. A multivariable linear regression model, incorporating VIF assessment for multicollinearity, was used to evaluate independent clinicopathological associations. Ordinal parameters were modeled as continuous variables to preserve statistical power. Multiple-testing correction (Benjamini–Hochberg procedure) was applied exclusively to high-dimensional exploratory analyses (PCA and GSEA), with associations considered statistically significant at a false discovery rate (FDR) of q < 0.05. Standard clinicopathological parameters and the targeted immune checkpoint subset were evaluated using nominal *p*-values. For each pathway-level PCA, Spearman’s ρ, nominal *p*-values, and adjusted q-values were reported. Effect sizes and 95% confidence intervals were calculated for all analyses and are detailed in Supplementary Table S1: Hodges–Lehmann estimator for Wilcoxon tests, Kendall’s tau for ordinal variables, Spearman’s rho for correlations, Beta coefficients for linear regression, and Hazard Ratios for Cox models.

All statistical analyses were conducted in RStudio (version 4.4.1). Two-tailed *p*-values < 0.05 were considered statistically significant.

## 5. Conclusions

This study reveals that COMP is significantly upregulated in CRC, positively correlating with advanced local tumor growth and regional lymph node involvement. Crucially, our protein-level analyses indicate that elevated COMP is closely associated with an immunosuppressive TME. High COMP levels are accompanied by restricted immune cell infiltration, reflected by a significant reduction in stromal tumor-infiltrating lymphocytes. Furthermore, COMP is linked to immune exhaustion, as indicated by significant positive correlations with the TIM-3/GAL9 checkpoint axis and SIGLEC9. The clear elevation of COMP in MSS tumors suggests that COMP-associated matrix remodeling may contribute to their resistance to immune infiltration. Consequently, COMP emerges as a predictive biomarker and a potential therapeutic target. Strategies aimed at disrupting COMP-associated fibrosis could offer a pathway to overcome immune exclusion in CRC.

## Figures and Tables

**Table 1:** Frequency of selected oncogenic mutations in the analyzed clinical cohort (*N* = 87).

Gene	Mutation Status	Number of Cases (*n*)	Frequency (%)
*KRAS*	Mutated	31	35.6
	Wild-type	56	64.4
*NRAS*	Mutated	12	13.8
	Wild-type	75	86.2
*BRAF*	Mutated	6	6.9
	Wild-type	81	93.1
*PIK3CA*	Mutated	6	6.9
	Wild-type	81	93.1
*AKT1*	Mutated	0	0.0
	Wild-type	87	100.0

**Table 2:** Correlation analysis between COMP expression and PCs derived from cytokine and immune checkpoint subsets. Abbreviations: COMP, Cartilage Oligomeric Matrix Protein; PC, principal component; TIM-3, T-cell immunoglobulin and mucin-domain containing-3; GAL9, galectin-9; B7-H4, B7 homolog 4; IL-2Ra, interleukin-2 receptor alpha chain; MIF, macrophage migration inhibitory factor; IFN-γ, interferon gamma; HGF, hepatocyte growth factor; IL-16, interleukin-16; IL-6, interleukin-6; IL-1β, interleukin-1 beta; IL-17, interleukin-17.

Cytokine Group/Process	Principal Component (Factor)	Key Molecules (Dominant Loadings)	Correlation Coefficient (R)	*p*-Value
Selected Immune Checkpoint Subset	Factor 1	TIM-3 (+), GAL9 (+), B7-H4 (−)	0.648	0.002
Interleukin receptor SHC signaling (Reactome HSA-912526)	Factor 2	IL-2Ra (−)	−0.460	0.042
Interleukin-12 signaling (Reactome HSA-9020591)	Factor 2	MIF (−), IFN-γ (+)	−0.513	0.021
Regulation of interleukin-6 production (GO:0032675)	Factor 2	HGF (+), IL-16 (+), IL-6 (+), IFN-γ (−), IL-1β (−)	0.438	0.050
Regulation of interleukin-12 production (GO:0032655)	Factor 3	IL-17 (+)	−0.495	0.027
Th17 cell differentiation (KEGG hsa04659)	Factor 3	IL-1β (+), IL-17 (+)	−0.572	0.008

**Table 3:** Baseline clinicopathological characteristics of the study cohort. Abbreviations: SD, standard deviation; TNM, tumor, node, metastasis; T, primary tumor; N, regional lymph node; M, distant metastasis; MSI, microsatellite instability; MSS, microsatellite stable.

Parameter	Female (*n* = 50)	Male (*n* = 57)	All Cases (*N* = 107)
Age (years, mean ± SD)	65.61 ± 9.76	63.78 ± 9.02	64.65 ± 9.38
T parameter, *n* (%)			
T1	0 (0.0%)	3 (5.3%)	3 (2.8%)
T2	9 (18.0%)	8 (14.0%)	17 (15.9%)
T3	32 (64.0%)	32 (56.1%)	64 (59.8%)
T4	9 (18.0%)	12 (21.1%)	21 (19.6%)
Unknown	0 (0.0%)	2 (3.5%)	2 (1.9%)
N parameter, *n* (%)			
N0	19 (38.0%)	25 (43.9%)	44 (41.1%)
N1	20 (40.0%)	23 (40.4%)	43 (40.2%)
N2	11 (22.0%)	9 (15.8%)	20 (18.7%)
M parameter, *n* (%)			
M0	41 (82.0%)	44 (77.2%)	85 (79.4%)
M1	9 (18.0%)	13 (22.8%)	22 (20.6%)
TNM stage, *n* (%)			
I	6 (12.0%)	7 (12.3%)	13 (12.1%)
II	12 (24.0%)	16 (28.1%)	28 (26.2%)
III	23 (46.0%)	22 (38.6%)	45 (42.1%)
IV	9 (18.0%)	12 (21.1%)	21 (19.6%)
Localization, *n* (%)			
Left	31 (62.0%)	37 (64.9%)	68 (63.6%)
Right	14 (28.0%)	15 (26.3%)	29 (27.1%)
Unknown	5 (10.0%)	5 (8.8%)	10 (9.3%)
MSI, *n* (%)			
MSI	5 (10.0%)	5 (8.8%)	10 (9.3%)
MSS	26 (52.0%)	27 (47.4%)	53 (49.5%)
Unknown	19 (38.0%)	25 (43.9%)	44 (41.1%)

**Table 4:** Summary of enriched pathways and processes. B7H3—B7 homolog 3 (CD276); B7H4—B7 homolog 4 (VTCN1); BasicFGF—basic fibroblast growth factor (FGF2); b-NGF—beta-nerve growth factor; CTACK—cutaneous T-cell-attracting chemokine (CCL27); Eotaxin—eotaxin-1 (CCL11); GAL9—galectin-9 (LGALS9); G-CSF—granulocyte colony-stimulating factor; GM-CSF—granulocyte-macrophage colony-stimulating factor; GO—Gene Ontology; GRO-a—growth-regulated oncogene alpha (CXCL1); HGF—hepatocyte growth factor; HHLA2—Human endogenous retrovirus-H long terminal repeat-associating protein 2; IFN-a2—interferon alpha-2; IFN-g—interferon gamma; IL-12p40—interleukin-12 subunit p40; IL-12p70—interleukin-12 subunit p70; IL-1Ra—interleukin-1 receptor antagonist; IL-2Ra—interleukin-2 receptor alpha chain; IP-10—interferon gamma-induced protein 10 (CXCL10); KEGG—Kyoto Encyclopedia of Genes and Genomes; LIF—leukemia inhibitory factor; MCP-1—monocyte chemoattractant protein-1 (CCL2); MCP-3—monocyte chemoattractant protein-3 (CCL7); M-CSF—macrophage colony-stimulating factor; MIF—macrophage migration inhibitory factor; MIG—monokine induced by gamma interferon (CXCL9); MIP-1a—macrophage inflammatory protein-1 alpha (CCL3); MIP-1b—macrophage inflammatory protein-1 beta (CCL4); PDGF-bb—platelet-derived growth factor-BB; RANTES—Regulated on Activation, Normal T Cell Expressed and Secreted (CCL5); SCF—stem cell factor; SCGF-b—stem cell growth factor-beta; SDF-1a—stromal cell-derived factor-1 alpha (CXCL12); SIGLEC9—sialic acid-binding Ig-like lectin 9; TIM-3—T-cell immunoglobulin and mucin-domain containing-3 (HAVCR2); TNF-a—tumor necrosis factor alpha; TNF-b—tumor necrosis factor beta (lymphotoxin-alpha); VEGFA—vascular endothelial growth factor A.

Process Name	Cytokines Involved	Origin
Positive regulation of immune system process	MIF, SCF, MCP1, SDF-1a, VEGFA, MCP3, MCSF, MIP-1a, IL-1a, IL-18, IL-6, RANTES, IL-5, TNF-b, LIF, IL-2, IL-1b, IL-7, IFN-g, IL-13, TNF-a, IL-10, IL-8, IL-4, IP-10, IL-15, IL-2Ra, IL-16, CTACK, IL-12p40, MIP-1b, IL-17	GO
Chemokine signaling pathway	IL-8, MCP1, SDF-1a, GRO-a, IP-10, RANTES, MIP-1a, CTACK, Eotaxin, MCP3, MIP-1b	KEGG
Positive regulation of lymphocyte migration	IP-10, SDF-1a, MIP-1a, CTACK, MIP-1b, MCP3, RANTES	GO
Macrophage chemotaxis	MCP1, IL-8, Eotaxin, MIG, IP-10, GRO-a, MIP-1b, MCP3, RANTES, IL-1b	GO
Regulation of cell population proliferation	CTACK, PDGF-bb, LIF, SCF, MIF, BasicFGF, IFN-g, IL-4, GM-CSF, G-CSF, IL-7, IL-3, MCSF, SDF-1a, SCGF-b, IL-2Ra, TNF-a, IL-6, IL-1b, IL-1a, IP-10, RANTES, IL-5, Eotaxin, IL-10, IL-2, IL-18, IL-15, IL-13, IL-9, TNF-b	KEGG
PI3K-Akt signaling pathway	IL-2Ra, bNGF, IL-2, IL-3, IL-4, SCF, MCSF, IFN-a2, HGF, G-CSF, IL-7, PDGF-bb, BasicFGF, IL-6, VEGFA	KEGG
Leukocyte activation	IL-4, IL-15, IFN-g, SCF, IL-2Ra, IL-8, MCSF, IL-13, IL-18, MIP-1a, RANTES, IL-10, GM-CSF, IL-9, IL-7, IFN-a2, IL-2, IL-6, TNF-a	GO
Inflammatory response	IL-9, CTACK, Eotaxin, MCP1, IFN-a2, IL-1Ra, IL-2Ra, IFN-g, IL-15, IL-1a, IL-6, IL-17, IL-4, MCP3, MIP-1a, IL-18, MIF, TNF-a, RANTES, MCSF, MIG, IL-1b, IL-5, IL-10, IL-8, IL-13, IP-10, MIP-1b	GO
Regulation of cell death	VEGFA, HGF, SDF-1a, bNGF, BasicFGF, SCF, IL-6, IL-7, G-CSF, IL-1a, IL-4, MCSF, IL-13, MIP-1a, IP-10, IL-1b, IL-9, IL-2, IFN-g, MCP1, TNF-b, GM-CSF, TNF-a, RANTES, IL-10, MIF	GO
RAF/MAP kinase cascade	PDGF-bb, IL-5, IL-2, BasicFGF, IL-2Ra, SCF, GM-CSF, HGF, IL-3	KEGG
Positive regulation of cytokine production	IL-9, IL-12p70, GM-CSF, IL-10, HGF, IL-2, IL-15, IL-1b, IL-18, IFN-g, IL-7, IL-4, TNF-a, IL-17, MIF, TNF-b, IL-16, IL-13, MIP-1a, IL-1a, IL-6	GO
Interleukin receptor SHC signaling	IL-2, IL-5, IL-3, GM-CSF, IL-2Ra	Reactome
Interleukin-12 signaling	MIF, IFN-g, IL-12p40, IL-12p70, IL-10	Reactome
Regulation of interleukin-6 production	HGF, IFN-g, IL-1a, IL-1b, IL-16, IL-6, TNF-a, IL-10, IL-17	GO
Regulation of interleukin-12 production	IFN-g, IL-12p40, IL-16, IL-10, IL-17	GO
Th17 cell differentiation	IL-2, IFN-g, IL-4, IL-1b, IL-2Ra, IL-6, IL-17	KEGG
NOD-like Receptor Signaling Pathway	IL-8, TNF-a, MCP1, IFN-a2, GRO-a, IL-6, IL-1b, RANTES, IL-18	KEGG
IL-17 Signaling Pathway	MCP1, IL-8, IL-1b, IL-4, IL-5, IL-6, IL-13, IL-17, GM-CSF, IFN-g, G-CSF, TNF-a, MCP3, Eotaxin, GRO-a, IP-10	KEGG
NF-kappa B signaling pathway	IL-1b, IL-8, GRO-a, SDF-1a, TNF-a, TNF-b, MIP-1b	KEGG
Regulation of angiogenesis	HGF, IL-1a, IL-1b, BasicFGF, Eotaxin, IP-10, IL-8, IL-6, TNF-a, IL-10	GO
Selected Immune Checkpoint Subset	TIM-3, GAL9, B7H3, HHLA2, SIGLEC9, B7H4	Custom analytical approach

## Data Availability

GSEA data: The FieldEffectCrc dataset [59]. Clinical and transcriptomic data from the Colorectal Adenocarcinoma (TCGA, PanCancer Atlas) dataset are publicly available via the cBioPortal for Cancer Genomics (https://www.cbioportal.org (accessed on 24 October 2025)). Experimental data can be shared upon request.

## Supplementary Materials

The following supporting information can be downloaded at: https://www.mdpi.com/article/10.3390/ijms27136032/s1.

## Abbreviations

The following abbreviations are used in this manuscript:

APC	Adenomatous polyposis coli
B7-H3	B7 homolog 3 protein (CD276)
B7-H4	B7 homolog 4 protein (VTCN1)
BasicFGF	Basic fibroblast growth factor (FGF2)
b-NGF	Beta-nerve growth factor
CMS	Consensus molecular subtypes
COMP	Cartilage oligomeric matrix protein
CTACK	Cutaneous T-cell-attracting chemokine (CCL27)
DAB	3,3′-diaminobenzidine
dMMR	Mismatch repair-deficient
ECM	Extracellular matrix
EGFR/Ras	Epidermal growth factor receptor/rat sarcoma virus
EMT	Epithelial–mesenchymal transition
FFPE	Formalin-fixed, paraffin-embedded
GAL9	Galectin-9
G-CSF	Granulocyte colony-stimulating factor
GM-CSF	Granulocyte-macrophage colony-stimulating factor
GO	Gene ontology
GRO-a	Growth-regulated oncogene alpha (CXCL1)
GSEA	Gene Set Enrichment Analysis
HGF	Hepatocyte growth factor
HHLA2	Human endogenous retrovirus-H long terminal repeat-associating protein 2
IFN-a2	Interferon alpha-2
IFN-γ	Interferon gamma
IL-12p40	Interleukin-12 subunit p40
IL-12p70	Interleukin-12 subunit p70
IL-1Ra	Interleukin-1 receptor antagonist
IL-2Ra	Interleukin-2 receptor alpha chain
IP-10	Interferon gamma-induced protein 10 (CXCL10)
KEGG	Kyoto Encyclopedia of Genes and Genomes
LIF	Leukemia inhibitory factor
M-CSF	Macrophage colony-stimulating factor
MCP-1	Monocyte chemoattractant protein-1 (CCL2)
MCP-3	Monocyte chemoattractant protein-3 (CCL7)
MIF	Macrophage migration inhibitory factor
MIG	Monokine induced by gamma interferon (CXCL9)
MIP-1a	Macrophage inflammatory protein-1 alpha (CCL3)
MIP-1b	Macrophage inflammatory protein-1 beta (CCL4)
MSI-H	Microsatellite instability-high
MSigDB	Molecular Signatures Database
NES	Normalized enrichment score
PCA	Principal Component Analysis
PDGF-bb	Platelet-derived growth factor-BB
pMRR	Mismatch repair proficient
RANTES	Regulated on activation, normal T cell expressed and secreted (CCL5)
SCF	Stem cell factor
SCGF-b	Stem cell growth factor-beta
SDF-1a	Stromal cell-derived factor-1 alpha (CXCL12)
SIGLEC9	Sialic acid-binding Ig-like lectin 9
SMAD4	SMAD family member 4 (DPC4)
TILs	Tumor-infiltrating lymphocytes
TIM-3	T-cell immunoglobulin and mucin-domain containing-3 (HAVCR2)
TME	Tumor microenvironment
TNF-α	Tumor necrosis factor alpha
TNF-b	Tumor necrosis factor beta (lymphotoxin-alpha)
VEGFA	Vascular endothelial growth factor A
