# Supplementary material for: Cartilage Oligomeric Matrix Protein (COMP) Correlates with Disease Progression, Selected Immune Checkpoint Molecules and SIGLEC9 in Colorectal Cancer
Source: Int J Mol Sci. 2026 Jul 5;27(13):6032. doi: 10.3390/ijms27136032 (PMC13362197; doi:10.3390/ijms27136032)
Supplement: Supplementary file 1 [file ijms-27-06032-s001.zip › ijms-4400030-supplementary.pdf]

Cartilage Oligomeric Matrix Protein (COMP) Correlates with Disease Progression, selected immune checkpoint molecules and SIGLEC9 in colorectal cancer

Supplementary materials

Table S1. Statistical parameters for COMP-related analyses across both cohorts. Effect sizes and 95% confidence intervals (CIs) were calculated using the Hodges-Lehmann estimator (Wilcoxon), Kendall's tau, Spearman's rho, multivariable linear regression (Beta), and Cox proportional hazards (HR). NA values indicate calculation failure due to group imbalance.

Analysis_Category	Variable	Test	Effect_Size	CI_Lower	CI_Upper	p_value
Clinicopathological Associations	T	Kendall	0.18	-0.00978	0.358	0.017888
Clinicopathological Associations	N	Kendall	0.21	0.0214	0.385	0.005688
Clinicopathological Associations	Stage	Kendall	0.233	0.0453	0.405	0.001632
Clinicopathological Associations	TILS	Kendall	-0.234	-0.456	0.0144	0.016904
Clinicopathological Associations	M	Wilcoxon	-0.234	-0.543	0.105	0.155907
Clinicopathological Associations	MSI	Wilcoxon	0.456	0.0208	1.1	0.003832
Mutational Status	KRAS (N=87)	Wilcoxon	0.0225	-0.274	0.381	0.71624
Mutational Status	NRAS (N=87)	Wilcoxon	0.17	-0.291	0.67	0.318597
Mutational Status	BRAF (N=87)	Wilcoxon	0.00975	-0.606	0.682	0.899995
Mutational Status	PIK3CA (N=87)	Wilcoxon	-0.592	-1.08	0.0595	0.074345
Immune Correlations	SIGLEC9	Spearman	0.312	0.0693	0.519	0.012885
Immune Correlations	TIM3	Spearman	0.273	0.0775	0.448	0.006872
Immune Correlations	GAL9	Spearman	0.262	0.0665	0.439	0.009401
Immune Correlations	HHLA2	Spearman	-0.245	-0.423	-0.0475	0.015743
Immune Correlations	B7H3	Spearman	0.0452	-0.159	0.246	0.665473
Immune Correlations	B7H4	Spearman	0.0455	-0.155	0.243	0.658204
Multivariable Linear Regression	(Intercept)	Multiple Regression (Beta)	0.294602	-0.56053	1.149736	0.493075
Multivariable Linear Regression	Stage	Multiple Regression (Beta)	0.193901	-0.02852	0.416319	0.086247
Multivariable Linear Regression	MSI	Multiple Regression (Beta)	-0.3193	-0.85123	0.21262	0.234317
Multivariable Linear Regression	TILS	Multiple Regression (Beta)	-0.12156	-0.33742	0.094291	0.26416
Clinicopathological Associations (TCGA)	T	Kendall	0.148	0.0684	0.226	4.24E-06

**Clinicopathological Associations (TCGA)**	**N**	**Kendall**	**0.147**	**0.0675**	**0.226**	**4.60E-06**
**Clinicopathological Associations (TCGA)**	**M**	**Wilcoxon**	**NA**	**NA**	**NA**	**0.231114**
**Clinicopathological Associations (TCGA)**	**Stage**	**Kendall**	**0.136**	**0.0549**	**0.215**	**1.47E-05**
**Immune Correlations (TCGA)**	**SIGLEC9**	**Spearman**	**0.52**	**0.458**	**0.576**	**2.70E-42**
**Immune Correlations (TCGA)**	**HAVCR2**	**Spearman**	**0.475**	**0.41**	**0.535**	**1.22E-34**
**Immune Correlations (TCGA)**	**LGALS9**	**Spearman**	**0.0394**	**-0.0413**	**0.12**	**0.338649**
**Immune Correlations (TCGA)**	**HHLA2**	**Spearman**	**-0.258**	**-0.332**	**-0.181**	**1.84E-10**
**Immune Correlations (TCGA)**	**CD276**	**Spearman**	**0.36**	**0.287**	**0.428**	**1.65E-19**
**Immune Correlations (TCGA)**	**VTCN1**	**Spearman**	**0.00842**	**-0.0722**	**0.0889**	**0.837962**
**Survival Analysis (TCGA)**	**KM - OS**	**Cox Regression**	**1.38**	**0.963**	**1.99**	**0.078038**
**Survival Analysis (TCGA)**	**KM - DSS**	**Cox Regression**	**2.03**	**1.24**	**3.31**	**0.003863**
**Survival Analysis (TCGA)**	**KM - DFS**	**Cox Regression**	**1.67**	**0.773**	**3.59**	**0.187716**
**Survival Analysis (TCGA)**	**KM - PFS**	**Cox Regression**	**1.44**	**1.04**	**1.99**	**0.028889**

Table S2. Eigenvalues and proportion of explained variance from the PCA of COMP and Selected Immune Checkpoint Subset.

**Principal Component**	**Standard Deviation**	**Eigenvalue**	**Variance (%)**	**Cumulative Variance (%)**
PC1 (Dim.1)	1.6307	2.6592	33.24	33.24
PC2 (Dim.2)	1.5032	2.2595	28.24	61.48
PC3 (Dim.3)	1.0627	1.1294	14.12	75.60

Table S3. Variable loadings for principal components derived from PCA of COMP and Selected Immune Checkpoint Subset.

Molecule	PC1 (Dim.1)	PC2 (Dim.2)	PC3 (Dim.3)
CTLA4	−0.3725	−0.6582	−0.1724
PD-L1	0.3727	0.0817	−0.8936
HHLA2	−0.3535	−0.6551	−0.2749
B7-H3	0.5541	0.6838	−0.1958
B7-H4	−0.6749	0.5225	0.2257
SIGLEC9	0.1791	−0.7179	0.1067
TIM-3	0.8166	−0.3365	0.2973
GAL9	0.8918	−0.1460	0.1912

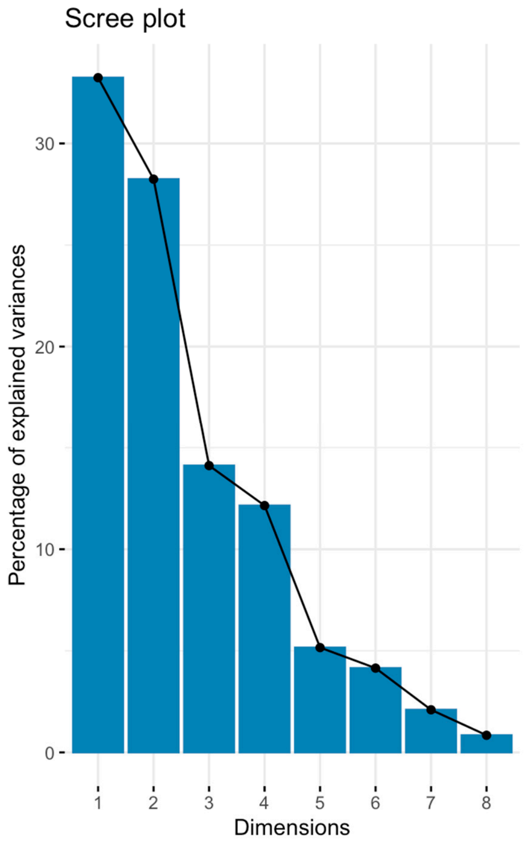

Figure S1. Scree plot of PCA for COMP and Selected Immune Checkpoint Subset molecules. Bars represent the percentage of explained variance for each principal component, and the connected line indicates the cumulative trend.

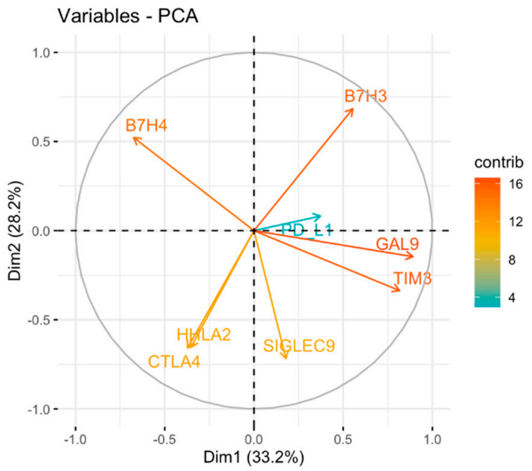

Figure S2. PCA biplot of Selected Immune Checkpoint Subset molecules in relation to COMP expression. The first two principal components are shown. Arrows represent variable loadings, and color intensity reflects contribution to the component structure.

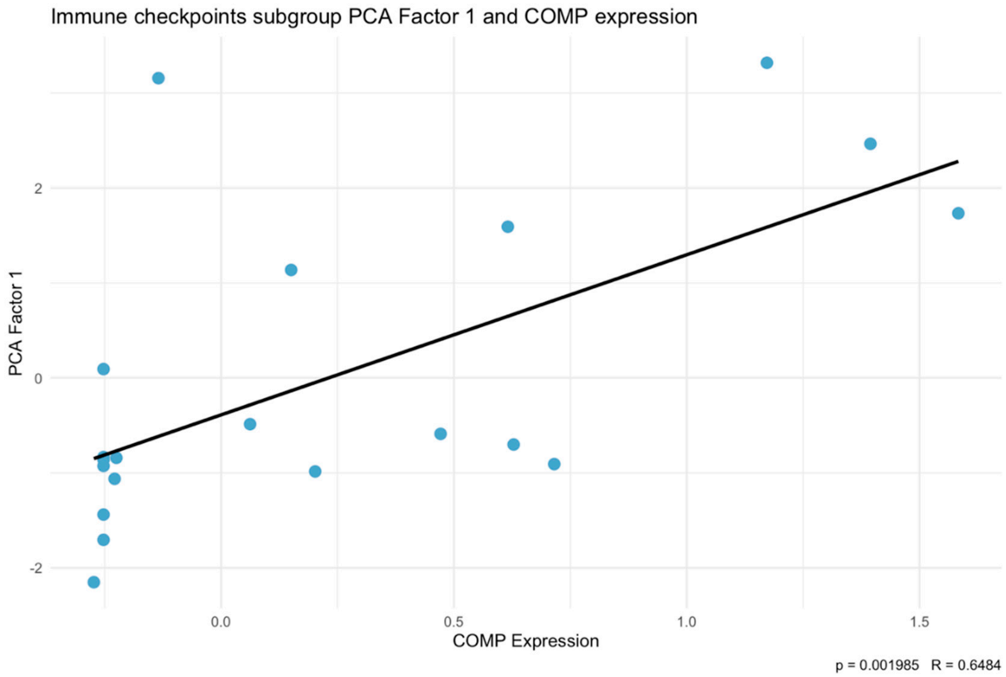

Figure S3. Correlation between PCA Factor 1 (Selected Immune Checkpoint Subset) and COMP expression. Each point represents an individual sample.

Table S4. PCA of COMP and Reactome HSA-912526 (Interleukin receptor SHC signaling). Eigenvalues and proportion of explained variance.

Principal Component	Standard Deviation	Eigenvalue	Variance (%)	Cumulative Variance (%)
PC1 (Dim.1)	1.9683	3.8742	77.48	77.48
PC2 (Dim.2)	0.7474	0.5586	11.17	88.66
PC3 (Dim.3)	0.6503	0.4229	8.46	97.11

Table S5. Variable loadings for the first three principal components derived from PCA of Reactome HSA-912526 (Interleukin receptor SHC signaling).

Cytokine	PC1 (Dim.1)	PC2 (Dim.2)	PC3 (Dim.3)
IL-2	0.9651	0.1292	0.0783
IL-5	0.8134	0.2671	−0.5082
IL-3	0.9731	0.0249	0.0826
GM-CSF	0.8935	0.1400	0.3749
IL-2Ra	0.7322	−0.6711	−0.1059

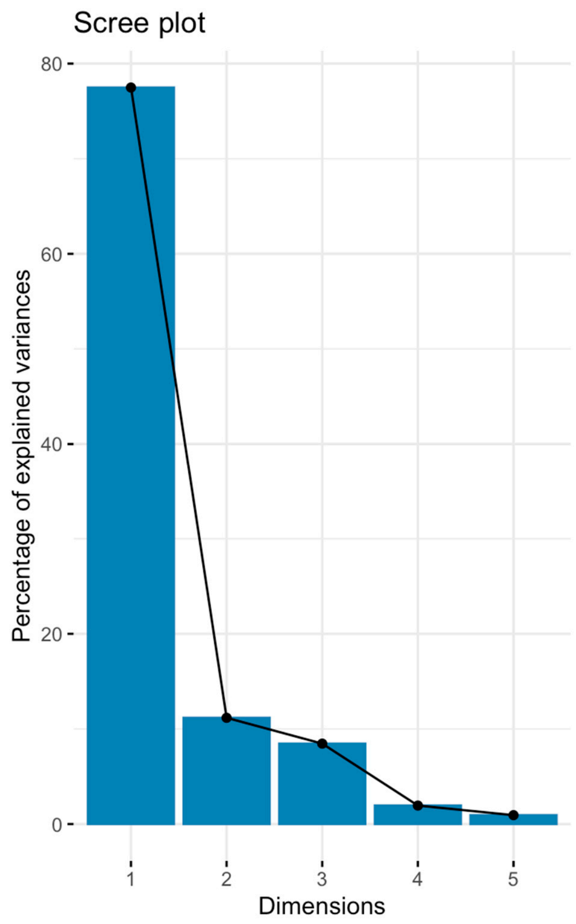

Figure S4. Scree plot of PCA for Reactome HSA-912526 (Interleukin receptor SHC signaling). Bars indicate the percentage of explained variance for each component.

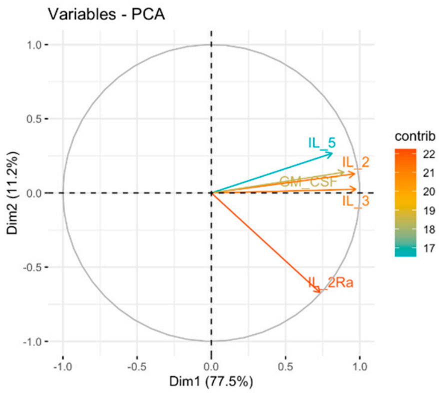

Figure S5. PCA biplot of cytokines included in Reactome HSA-912526 (Interleukin receptor SHC signaling). The first two principal components are shown.

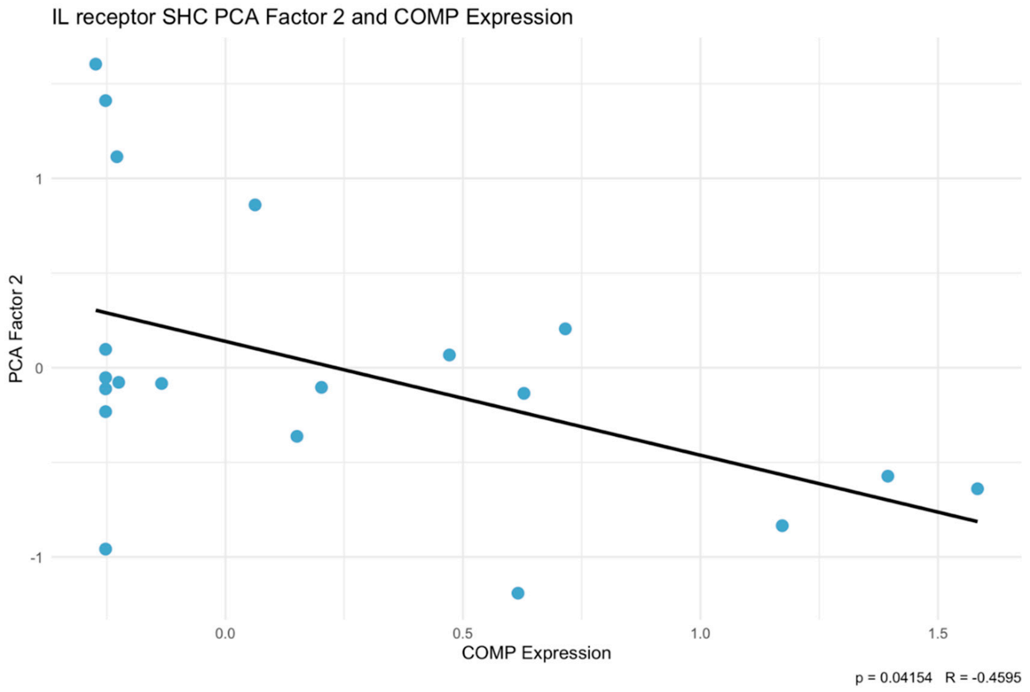

Figure S6. Correlation between PCA Factor 2 (Interleukin receptor SHC signaling subset) and COMP expression. Each point represents an individual sample.

Table S6. PCA of COMP and Reactome HSA-9020591 (Interleukin-12 signaling). Eigenvalues and proportion of explained variance.

Principal Component	Standard Deviation	Eigenvalue	Variance (%)	Cumulative Variance (%)
PC1 (Dim.1)	1.7141	2.9380	58.76	58.76
PC2 (Dim.2)	1.0606	1.1248	22.50	81.26
PC3 (Dim.3)	0.8580	0.7362	14.72	95.98

Table S7. Variable loadings for the first three principal components derived from PCA of Reactome HSA-9020591 (Interleukin-12 signaling).

Cytokine	PC1 (Dim.1)	PC2 (Dim.2)	PC3 (Dim.3)
MIF	−0.2379	−0.8036	0.5454
IFN-γ	0.4709	0.6072	0.6359
IL-12p40	0.9000	−0.2862	−0.1492
IL-12p70	0.9549	−0.1485	−0.0946
IL-10	0.9685	−0.0803	0.0567

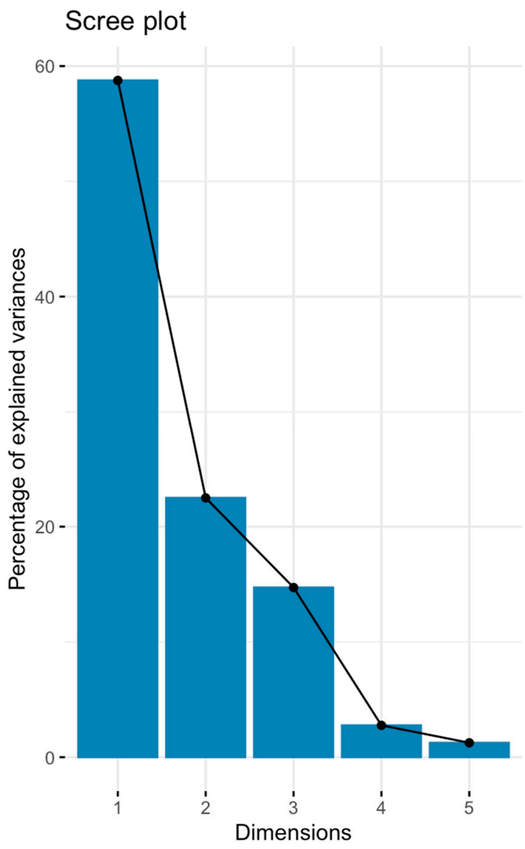

Figure S7. Scree plot of PCA for Reactome HSA-9020591 (Interleukin-12 signaling). Bars represent the percentage of explained variance for each principal component.

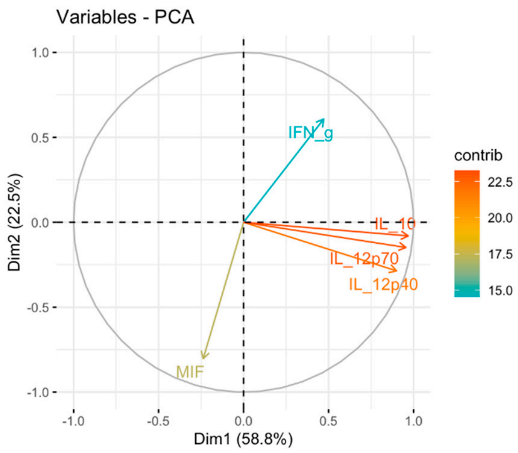

Figure S8. PCA biplot of cytokines included in Reactome HSA-9020591 (Interleukin-12 signaling). The first two principal components are shown.

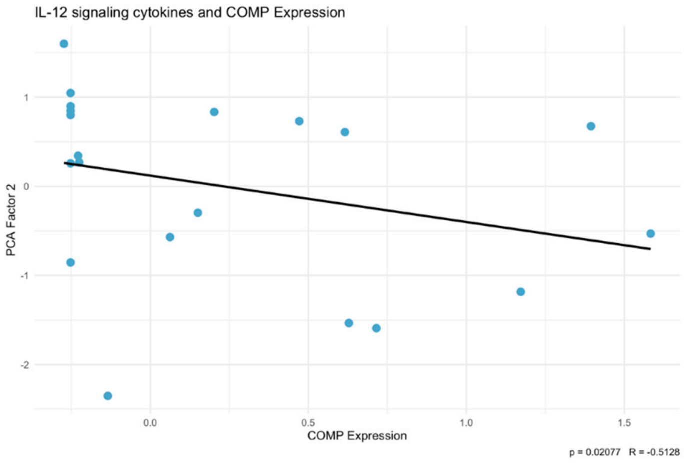

Figure S9. Correlation between PCA Factor 2 (Interleukin-12 signaling subset) and COMP expression. Each point represents an individual sample.

Table S8. PCA of COMP and GO:0032675 (Regulation of interleukin-6 production). Eigenvalues and proportion of explained variance.

Principal Component	Standard Deviation	Eigenvalue	Variance (%)	Cumulative Variance (%)
PC1 (Dim.1)	1.9348	3.7436	41.60	41.60
PC2 (Dim.2)	1.5698	2.4643	27.38	68.98
PC3 (Dim.3)	1.0368	1.0750	11.94	80.92

Table S9. Variable loadings for the first three principal components derived from PCA of GO:0032675 (Regulation of interleukin-6 production).

Cytokine	PC1 (Dim.1)	PC2 (Dim.2)	PC3 (Dim.3)
HGF	0.3796	0.7929	0.1266
IFN-γ	0.4382	−0.6027	0.5557
IL-1α	0.8591	−0.3109	0.1068
IL-1β	0.5837	−0.5658	−0.3270
IL-16	0.3163	0.7671	0.0153
IL-6	0.5180	0.6369	−0.1884
TNF-α	0.9241	0.0412	0.1178
IL-10	0.8947	0.0856	0.2189
IL-17	0.5532	−0.2290	−0.7309

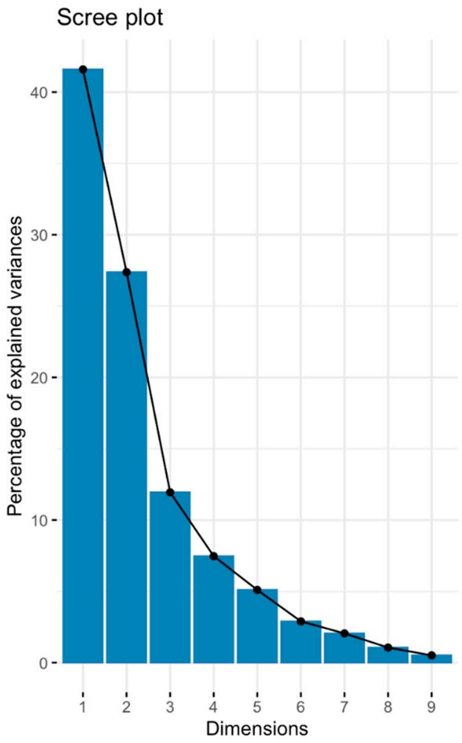

Figure S10. Scree plot of PCA for GO:0032675 (Regulation of interleukin-6 production). Bars indicate the percentage of explained variance for each component.

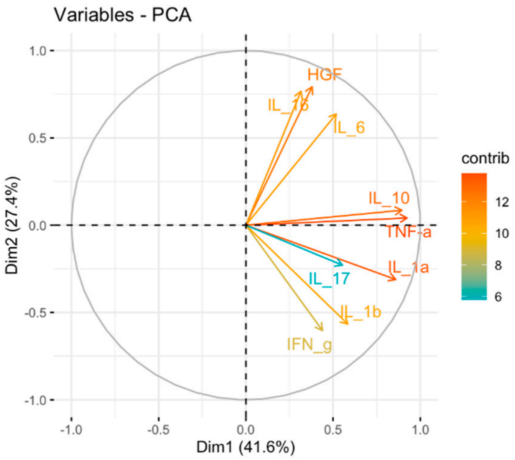

Figure S11. PCA biplot of cytokines included in GO:0032675 (Regulation of interleukin-6 production). The first two principal components shown.

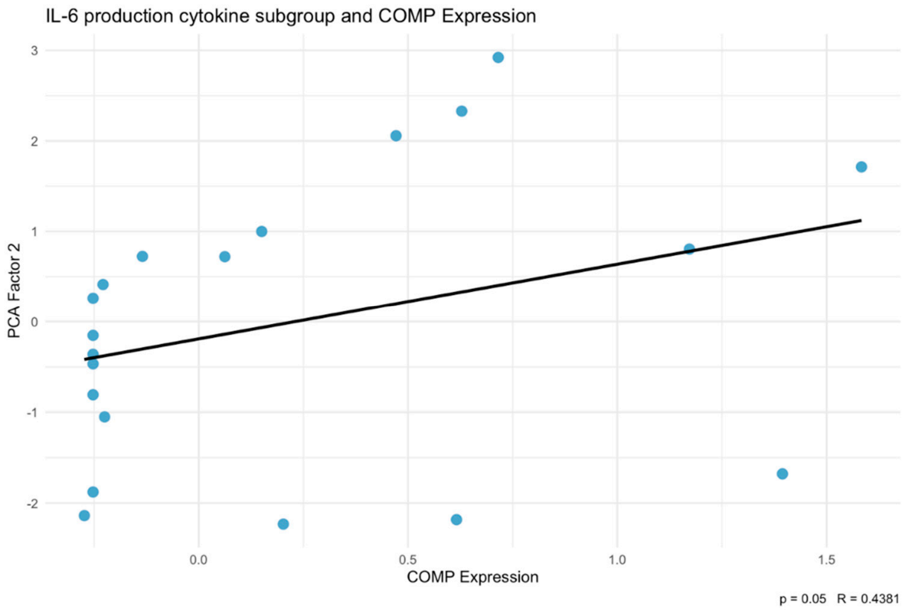

Figure S12. Correlation between PCA Factor 2 (IL-6 production cytokine subset) and COMP expression. Each point represents an individual sample.

Table S10. PCA of COMP and GO:0032655 (Regulation of interleukin-12 production). Eigenvalues and proportion of explained variance.

Principal Component	Standard Deviation	Eigenvalue	Variance (%)	Cumulative Variance (%)
PC1 (Dim.1)	1.5043	2.2629	45.26	45.26
PC2 (Dim.2)	1.1326	1.2828	25.66	70.91
PC3 (Dim.3)	0.9779	0.9562	19.12	90.04

Table S11. Variable loadings for the first three principal components derived from PCA of GO:0032655 (Regulation of interleukin-12 production).

Cytokine	PC1 (Dim.1)	PC2 (Dim.2)	PC3 (Dim.3)
IFN-γ	0.3514	−0.8321	−0.2729
IL-12p40	0.9047	0.0997	−0.1455
IL-16	0.4430	0.7497	−0.2915
IL-10	0.9650	−0.1356	−0.0317
IL-17	0.4399	0.0019	0.8801

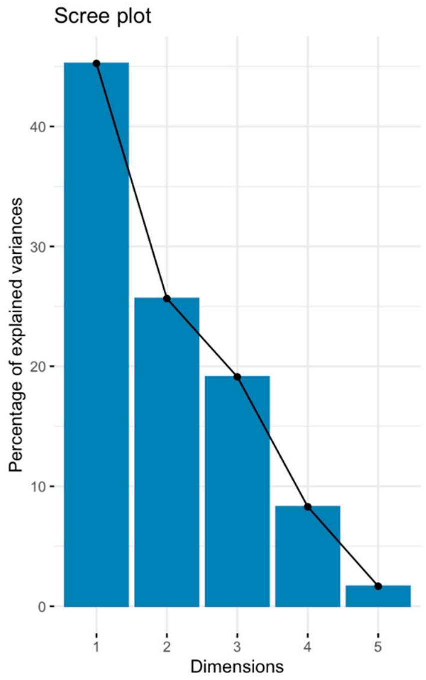

Figure S13. Scree plot of PCA for GO:0032655 (Regulation of interleukin-12 production). Bars represent the percentage of explained variance for each principal component.

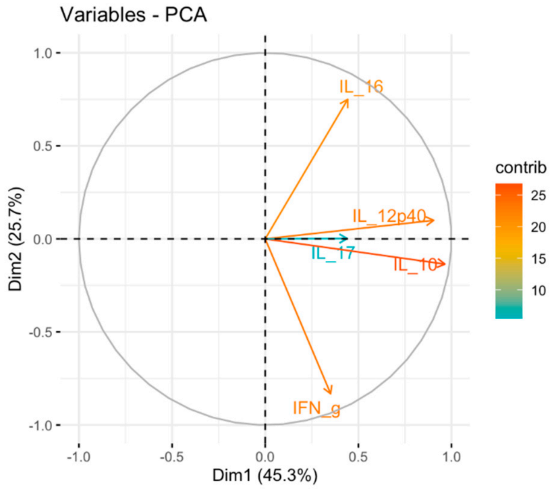

Figure S14. PCA biplot of cytokines included in GO:0032655 (Regulation of interleukin-12 production). The first two principal components are shown.

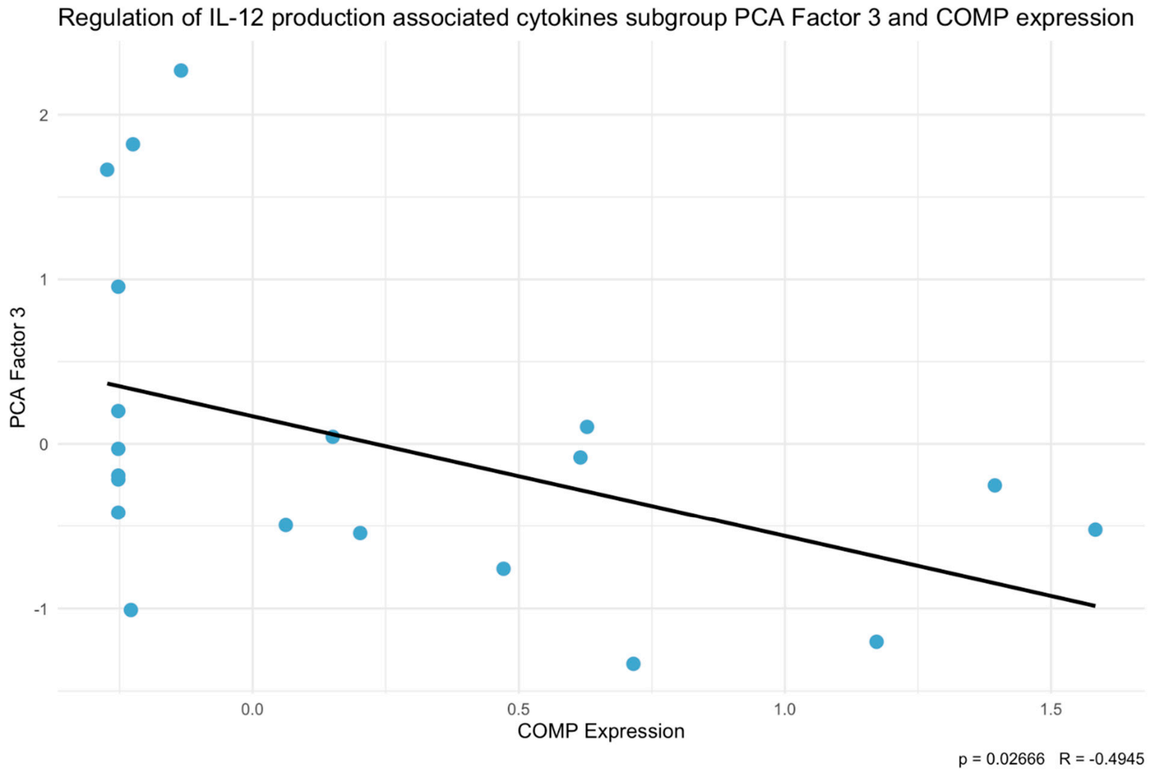

Figure S15. Correlation between PCA Factor 3 (Regulation of interleukin-12 production subset) and COMP expression. Each point represents an individual sample.

Table S12. PCA of COMP and KEGG hsa04659 (Th17 cell differentiation). Eigenvalues and proportion of explained variance.

Principal Component	Standard Deviation	Eigenvalue	Variance (%)	Cumulative Variance (%)
PC1 (Dim.1)	1.7855	3.1880	45.54	45.54
PC2 (Dim.2)	1.1085	1.2288	17.55	63.10
PC3 (Dim.3)	1.0546	1.1122	15.89	78.99

Table S13. Variable loadings for the first three principal components derived from PCA of KEGG hsa04659 (Th17 cell differentiation).

Cytokine	PC1 (Dim.1)	PC2 (Dim.2)	PC3 (Dim.3)
IL-2	0.8118	0.1070	−0.3027

Cytokine	PC1 (Dim.1)	PC2 (Dim.2)	PC3 (Dim.3)
IFN-γ	0.5357	−0.7621	0.0409
IL-4	0.8361	0.0757	−0.3994
IL-1β	0.6326	−0.1672	0.6970
IL-2Ra	0.8438	−0.0760	−0.2360
IL-6	0.3243	0.6825	−0.0174
IL-17	0.5706	0.3624	0.5636

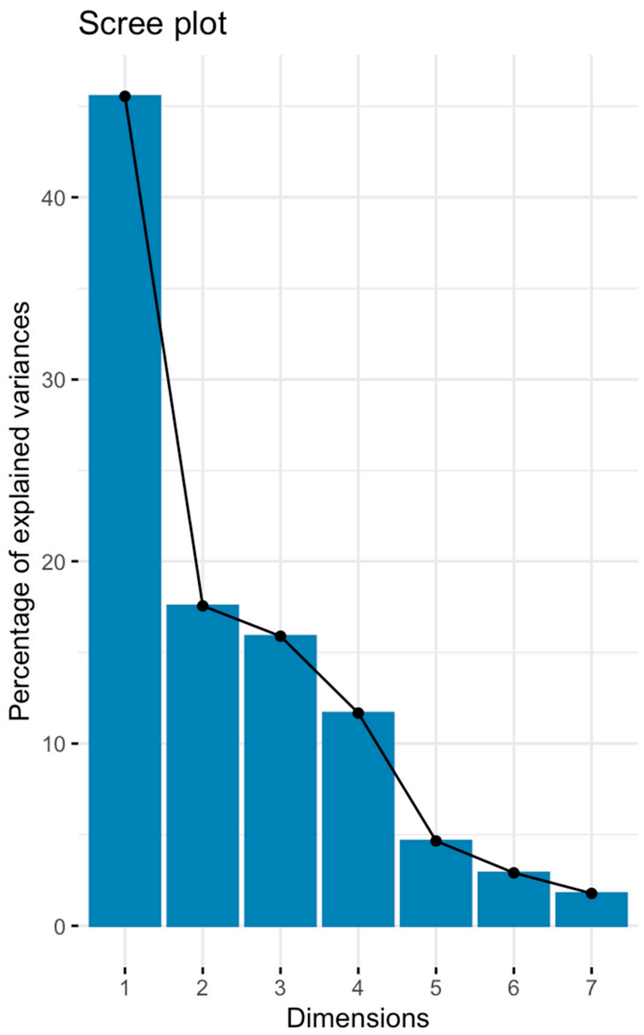

Figure S16. Scree plot of PCA for KEGG hsa04659 (Th17 cell differentiation). Bars indicate the percentage of explained variance for each component.

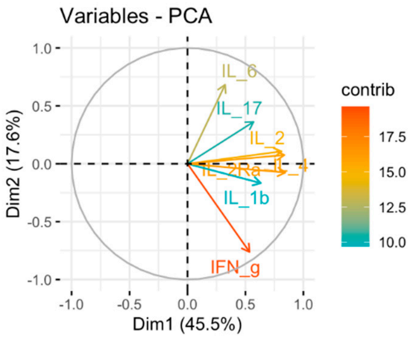

Figure S17. PCA biplot of cytokines included in KEGG hsa04659 (Th17 cell differentiation). The first two principal components are shown.

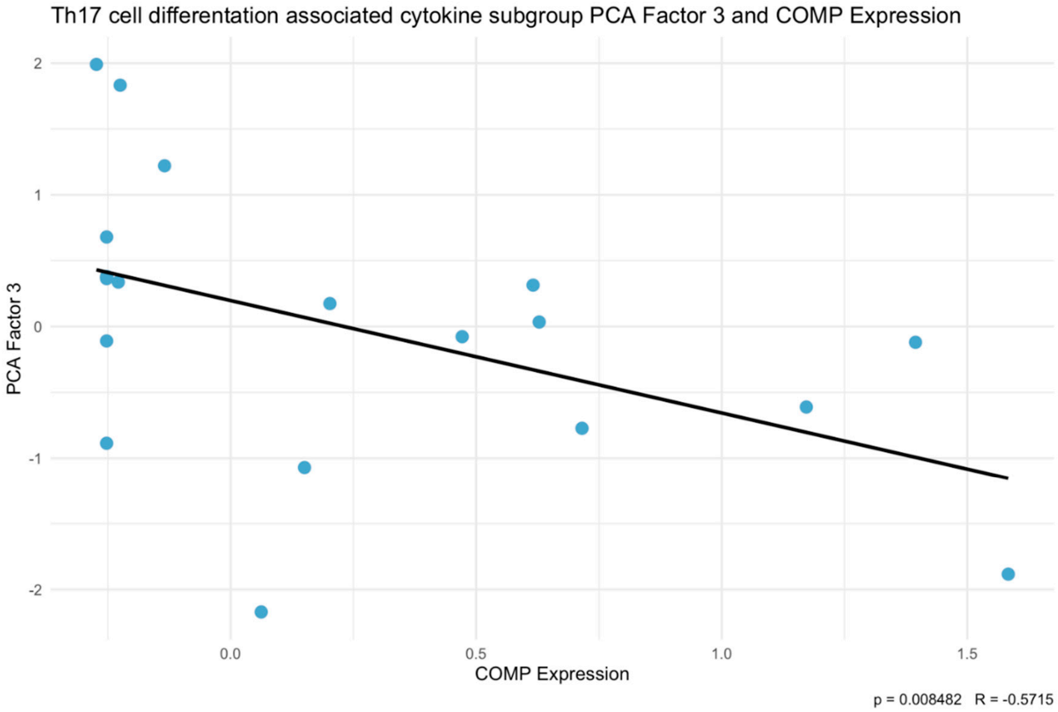

Figure S18. Correlation between PCA Factor 3 (Th17 differentiation cytokine subset) and COMP expression. Each point represents an individual sample.

COMP and GSEA

Table S14. Gene Set Enrichment Analysis (GSEA) of Hallmark pathways comparing COMP-high and COMP-low tumors (FDR < 0.05). Positive normalized enrichment scores (NES) indicate enrichment in COMP-high samples, whereas negative NES values indicate enrichment in COMP-low samples.

Pathway	NES	p-value	FDR (padj)	Gene Set Size	Enrichment
HALLMARK_HEME_METABOLISM	−1.60	0.000101	0.00102	197	Down-regulated
HALLMARK_XENOBIOTIC_METABOLISM	−1.65	0.000101	0.00102	200	Down-regulated
HALLMARK_ADIPOGENESIS	−1.91	0.000101	0.00102	200	Down-regulated
HALLMARK_FATTY_ACID_METABOLISM	−1.92	0.000102	0.00102	157	Down-regulated
HALLMARK_OXIDATIVE_PHOSPHORYLATION	−2.29	0.000101	0.00102	200	Down-regulated
HALLMARK_PEROXISOME	−1.68	0.000212	0.00177	103	Down-regulated
HALLMARK_ESTROGEN_RESPONSE_LATE	−1.50	0.000304	0.00217	200	Down-regulated
HALLMARK_BILE_ACID_METABOLISM	−1.58	0.000527	0.00329	111	Down-regulated
HALLMARK_ANGIOGENESIS	2.38	0.000627	0.00348	36	Up-regulated
HALLMARK_MYC_TARGETS_V2	1.84	0.000892	0.00446	58	Up-regulated
HALLMARK_COAGULATION	1.87	0.00303	0.0138	138	Up-regulated
HALLMARK_KRAS_SIGNALING_DN	−1.38	0.00416	0.0173	200	Down-regulated
HALLMARK_EPITHELIAL_MESENCHYMAL_TRANSITION	3.23	0.00694	0.0204	200	Up-regulated
HALLMARK_INFLAMMATORY_RESPONSE	1.53	0.00694	0.0204	200	Up-regulated
HALLMARK_APICAL_JUNCTION	1.42	0.00694	0.0204	200	Up-regulated
HALLMARK_MYC_TARGETS_V1	1.39	0.00694	0.0204	200	Up-regulated
HALLMARK_ANDROGEN_RESPONSE	−1.45	0.00885	0.0246	101	Down-regulated
HALLMARK_HEDGEHOG_SIGNALING	1.59	0.0138	0.0331	36	Up-regulated
HALLMARK_E2F_TARGETS	1.34	0.0139	0.0331	200	Up-regulated
HALLMARK_KRAS_SIGNALING_UP	1.33	0.0139	0.0331	200	Up-regulated
HALLMARK_PANCREAS_BETA_CELLS	−1.50	0.0146	0.0332	40	Down-regulated
HALLMARK_WNT_BETA_CATENIN_SIGNALING	1.54	0.0175	0.0381	42	Up-regulated

Table S15. Sample sizes across all analyses. Variations in the evaluated cohort size (N) are due to limited clinical data or tissue availability.

Analytical Approach	Variables / Targets Evaluated	Sample Size (N)
Protein expression profiling	COMP (Tumor vs. Matched Margins)	N = 107
Clinicopathological associations	N, M, Overall Stage, TILs	N = 107
	T stage	N = 105 (2 unknown)
Microsatellite Instability (MSI)	MSI status	N = 63

Multivariable Regression Model	COMP, Stage, MSI, TILs	N = 61
Mutational profiling	*KRAS*, *NRAS*, *BRAF*, *PIK3CA*, *AKT1*	N = 87
Immune Checkpoint profiling	SIGLEC9, TIM3, GAL9, HHLA2, B7H3, B7H4	N = 97
Multiplex Cytokine Array (PCA)	48 cytokines, chemokines, and growth factors	N = 54
TCGA Transcriptomic Analysis	Overall Survival (OS), Progression-Free Survival (PFS)	N = 588
	Disease-Specific Survival (DSS)	N = 567
	Disease-Free Survival (DFS)	N = 222
Gene Set Enrichment Analysis (GSEA)	FieldEffectCrc dataset	N = 311
